# Interactions among rooting traits for deep water and nitrogen uptake in upland and lowland ecotypes of switchgrass (*Panicum virgatum* L.)

**DOI:** 10.1093/jxb/erab437

**Published:** 2021-10-04

**Authors:** Marcus Griffiths, Xueyan Wang, Kundan Dhakal, Haichao Guo, Anand Seethepalli, Yun Kang, Larry M York

**Affiliations:** 1 Noble Research Institute, LLC, 2510 Sam Noble Parkway, Ardmore, OK 73401, USA; 2 Biosciences Division and Center for Bioenergy Innovation, Oak Ridge National Laboratory, Oak Ridge, TN 37831, USA; 3 CSIRO Agriculture and Food, Australia

**Keywords:** Abiotic stress, deep rooting, mesocosm, nitrogen, partitioning, plasticity, strategies, switchgrass, tolerance, water

## Abstract

The response of plant growth and development to nutrient and water availability is an important adaptation for abiotic stress tolerance. Roots need to intercept both passing nutrients and water while foraging into new soil layers for further resources. Substantial amounts of nitrate can be lost in the field when leaching into groundwater, yet very little is known about how deep rooting affects this process. Here, we phenotyped root system traits and deep ^15^N nitrate capture across 1.5 m vertical profiles of solid media using tall mesocosms in switchgrass (*Panicum virgatum* L.), a promising cellulosic bioenergy feedstock. Root and shoot biomass traits, photosynthesis and respiration measures, and nutrient uptake and accumulation traits were quantified in response to a water and nitrate stress factorial experiment for switchgrass upland (VS16) and lowland (AP13) ecotypes. The two switchgrass ecotypes shared common plastic abiotic responses to nitrogen (N) and water availability, and yet had substantial genotypic variation for root and shoot traits. A significant interaction between N and water stress combination treatments for axial and lateral root traits represents a complex and shared root development strategy for stress mitigation. Deep root growth and ^15^N capture were found to be closely linked to aboveground growth. Together, these results represent the wide genetic pool of switchgrass and show that deep rooting promotes nitrate capture, plant productivity, and sustainability.

## Teaser

Two main ecotypes of switchgrass have both shared and different root responses to varying water and nitrogen conditions, with deep rooting shown to be closely linked to aboveground growth.

## Introduction

The root system of a plant serves multiple important roles, from structural stability in the soil to resource foraging for water and nutrients. The spatial and temporal arrangement of roots in the soil (broadly referred to as root system architecture) can greatly affect the interception and subsequent uptake of soil resources. Root growth and development are highly responsive to both the environmental conditions and the plant’s resource requirements. Greater knowledge of this dynamic process in plants is important to characterize ecological adaptations and breed for beneficial adaptations enabling more resource-efficient plant varieties.

Water and nitrogen (N) are the two most frequently limiting resources in agriculture, affecting plant growth and yield. Both water and nitrate-N are highly mobile in the soil profile, which means plants have a limited opportunity to acquire these resources. Plant adaptive responses help mitigate such abiotic stresses through changes in growth and development in response to the plant’s nutritional requirements and the environment. Deep rooting is regarded as a beneficial trait for plant productivity and abiotic stress mitigation by expanding the vertical soil profile explored, effectively increasing deep soil resources available to the plant (reviewed by Thorup-Kristensen *et al.*, 2020). Increased root length at depth also enables plants to capture water and nitrate that otherwise would be lost through deep soil water movement and leaching. Deep rooting traits in turn are postulated to reduce the environmental damage caused by the leaching of nutrients into groundwater (Kumar and Goh, 2002; Foulkes *et al.*, 2009). Increasingly, the importance of deep roots has been acknowledged for the sequestration of carbon into stable soil organic carbon pools (Poirier *et al.*, 2018).

As the spatial–temporal distribution of soil resources is dynamic and variable across environments, it is also important to understand the trade-offs involved in the root spatial allocation (reviewed in van der Bom *et al.*, 2020). However, roots are challenging to phenotype and evaluate quantitatively as they are the hidden half of the plant, and relatively little is known about root growth, nutrient capture, and root longevity of perennial crops. At present, root evaluation at depth is often conducted by soil coring methods in the field, with subsequent root washing and image analysis or quantitative PCR for DNA abundance used for quantifying root length or mass (Kristensen and Thorup-Kristensen, 2004; Heuermann *et al.*, 2019), non-destructively using minirhizotrons or ingrowth cores that can be coupled with stable isotope tracing (Ehleringer and Dawson, 1992; Chen *et al.*, 2019; Svane *et al.*, 2019; Han *et al.*, 2020), or under more controlled set-ups using large rhizotrons (Nagel *et al.*, 2012; Ytting *et al.*, 2014) or large mesocosms (Saengwilai *et al.*, 2014; Zhan *et al.*, 2015; Guo and York, 2019).

Switchgrass (*Panicum virgatum* L.) is a C_4_, warm-season perennial grass that is native to North America and has an extensive and deep root system with recorded rooting depths of 330cm in field trials (Ma *et al.*, 2000). As with many prairie grasses, switchgrass develops rhizomes which are underground, stem-derived organs that provide plants with the ability to grow clonally and regrow after disturbance in the soil (Weaver, 1954; Freschet *et al.*, 2021). Switchgrass is found across a diverse geographical range from Canada to Central America, and has a promising utility as a cellulosic bioenergy feedstock. Switchgrass has low input requirements, so is ideal for growth on marginal lands. In addition, switchgrass is reported to provide ecosystem services with an enhancement of soil organic matter, reduction in soil erosion, and associative N fixation (Gilley *et al.*, 2000; Lai *et al.*, 2018; Roley *et al.*, 2019). Switchgrass can be divided into two main ecotypes, upland and lowland, which are estimated to have diverged 0.7–1.0 million years ago (Morris *et al.*, 2011). The ecotype divergence in switchgrass is hypothesized to be through climate-associated adaptation, with the upland ecotype found in more northern latitudes and across drier precipitation gradients than the lowland ecotype (Lovell *et al.*, 2021). The upland ecotype has also been found to be generally smaller with a greater number of tillers and an earlier flowering time (Milano *et al.*, 2016; Singer *et al.*, 2019). As the ecotypes are diverse, each has its own beneficial breeding potential with different environmental adaptation and pathogen resistance (Milano *et al.*, 2016). For switchgrass adoption as a bioenergy feedstock, the biomass yield will have to be maximized in a sustainable manner, which requires a greater understanding of the interactions among environment, ecotype, and soil dynamics (Lemus *et al.*, 2014).

In-depth characterization of the physiological and morphological differences of the main switchgrass ecotypes is important to understand the functional adaptations to resource capture and to characterize the differences in abiotic stress tolerance. The aim of this study was to characterize and compare the root systems of the representative upland and lowland ecotypes of switchgrass and the root adaptive responses to water and N stress. To achieve this, we set up a tall mesocosm greenhouse study with water and N factorial stresses using clones of representative upland and lowland cultivars and evaluated the vertical distribution of the root system across 150cm depth along with other physiological characteristics.

## Materials and methods

### Plant materials and experiment design

Clones derived from two contrasting genotypes of switchgrass, AP13 and VS16, were used in this study to represent the two ecotypes. AP13 is a clone derived from the lowland cultivar ‘Alamo’, which is the source of the switchgrass reference genome, and VS16 is a clone derived from the upland cultivar ‘Summer’. Mapping populations have been derived from these two ecotypes (Milano *et al.*, 2016), which highlights their importance for switchgrass research. Recently emerged tillers from well-established plants were pulled apart by hand, and one tiller consisting of a small shoot and root system was transplanted per mesocosm at the start of the experiment. The mesocosm experiment was conducted in a greenhouse from 30 September 2019 to 22 January 2020 at the Noble Research Institute, LLC, Ardmore, OK, USA (34°11ʹN, 97°5ʹW; elevation 268 m). The greenhouse conditions were set to a 15/7h day/night cycle at 24/21 °C with an average photosynthetically active radiation (PAR) reading of 150 μmol m^−2^ s^−1^ provided with supplemental lighting. Monthly averages for greenhouse conditions are provided in Supplementary Table S1. The mesocosm experiment was arranged as a randomized complete block design, replicated five times with a 2×2×2 factorial arrangement of treatments. The factors were two levels of N supply (high- and low-N, HN and LN), two watering levels (well-watered and drought-stressed, WW and DS), and two ecotypes (upland and lowland). The treatment combinations are hereafter referred to as HN/WW, LN/WW, HN/DS, and LN/DS.

### Mesocosm preparation

The media mixture used in the mesocosm study mimics mineral soil and consisted of sand, vermiculite, and perlite which was mixed using a cement mixer. By volume basis, the mixture constituents used were 50% medium size (0.3–0.5mm) premium sand (Quikrete, GA, USA), 40% premium grade vermiculite (Sun Gro Horticulture, Agawam, MA, USA), and 10% perlite (Ambient Minerals, AR, USA). The gravimetric water content of the media mixture at mesocosm filling and before watering was 2.5%, as determined by oven-drying 20g of medium at 105 °C for 48h (Equation 1) (Rowell, 1994).


$$\emptyset g=\left(\frac{\text{Wet soil mass}-\text{Dry soil mass}}{\text{Dry soil mass}}\right)$$ (1)

**Fig. 1.**
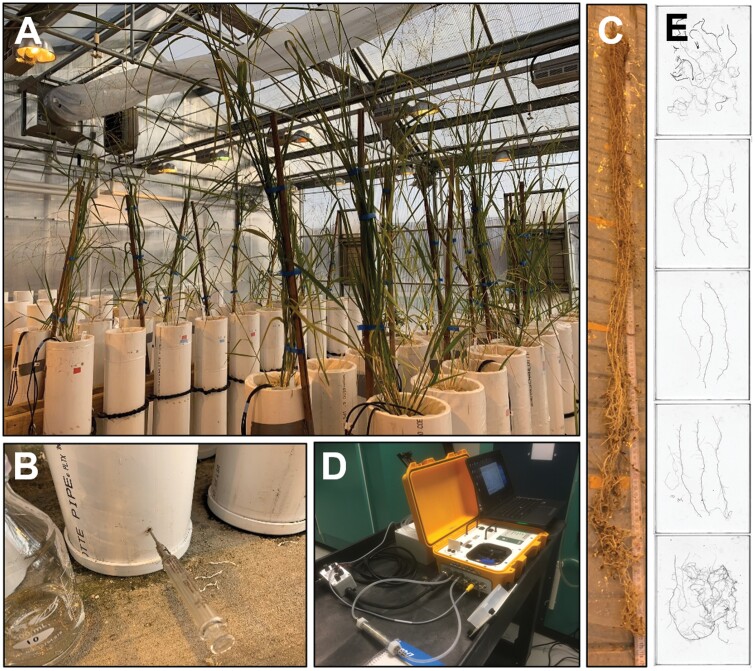
Switchgrass mesocosm experiment design. (A) Upland and lowland ecotypes were grown in tall mesocosms under factorial nitrogen and water stress conditions HN/WW, LN/WW, HN/DS, and LN/DS. (B) ^15^N was injected into the deepest layer of the mesocosms 24h before the shoot material was harvested. (C) The medium was carefully washed away, and the root system was cut into 30cm layers which were used for (D) instantaneous root respiration analysis using an LI-8100 with custom chambers, and (E) root feature determination by root scanning and image analysis using RhizoVision Explorer.

The mesocosms used in this study consisted of polyvinyl chloride (PVC) pipe cut to size 15.24cm (internal diameter)×152.4cm (height) with a flat-bottom PVC cap (IPS Corporation, Collierville, TN, USA), and lined with a seamless heavy-duty (6 mil) poly tubing (Uline, WI, USA) (Fig. 1A). The mesocosms were evenly filled from the top of the column with 28 liters of the air-dry medium, resulting in an approximate bulk density of 1.1g cm^–3^. Three days before transplanting, the mesocosms were irrigated from the top with 6 liters of nutrient solution. Half of the mesocosms received a zero N half-strength Hoagland’s solution for LN treatment, resulting in a severe N stress with only residual N in the media mixture as an N source at a concentration relevant for high affinity transport (Glass *et al.*, 1992). The other half of the mesocosms received a high N half-strength Hoagland’s solution (6mM NO_3_-N) for HN treatment. The high N solution was composed of (in µM) 500 KH_2_PO_4_, 5700 KNO_3_, 300 NH_4_NO_3_, 2000 CaCl_2_, 1000 MgSO_4_, 46 H_3_BO_3_, 7 ZnSO_4_·7H_2_O, 9 MnCl_2_·4H_2_O, 0.32 CuSO_4_·5H_2_O, 0.114 (NH_4_)_6_Mo_7_O_24_·4H_2_O, and 150 Fe(III)-EDTA (C_10_H_12_N_2_NaFeO_8_). For the zero N solution, KNO_3_ and NH_4_NO_3_ were replaced with 5700 µM KCl.

The residual N levels of the starting medium before the nutrient application was determined to be 1.74 μg N g^–1^ medium by ion chromatography. A 20g aliquot of medium was first added to 50ml of 0N half-strength Hoagland’s solution and then shaken for 30min at 150rpm. After shaking, the sample was left undisturbed for 5min for the particles to settle, and then 5ml of the supernatant was centrifuged at 10000rpm for 5min in a 15ml falcon tube. The ion concentrations of the collected supernatant samples were then determined using a Thermo Scientific ICS-5000+ ion chromatographic system employing 500 μl of the sample (Thermo Fisher Scientific, MA, USA).

### Mesocosm greenhouse growth conditions

One tiller of a clone was transplanted per mesocosm; the mesocosms were watered with the respective nutrient solution three times a week with 200ml added from the top at each watering. For all mesocosms at sowing, the average gravimetric water content across the whole mesocosm was 22% with initially more water at the top from watering. After 4 weeks of growth in the mesocosms, half of the mesocosms were subjected to drought stress, receiving no more watering for the rest of the experiment. The well-watered mesocosms continued to be watered three times a week with 200ml of double-deionized water instead of nutrient solution. A subset of the mesocosms were weighed three times a week and the watering regime was sufficient to maintain the well-watered mesocosms at a constant weight. At the end of the experiment, the drought-stressed mesocosms had a gravimetric water content ranging from 16% in the first 30cm layer to 28% in the deepest 30cm layer, with more water availability at depth (Table 1). The gravimetric water content of the well-watered mesocosms ranged from 27% in the first 30cm layer to 34% in the bottom layer (Table 1). Averaged across the whole mesocosm, the gravimetric water content for the water-stressed and well-watered mesocosms were 23% and 29%, respectively.

**Table 1.** Gravimetric water content (%) of mesocosms determined at the end of the greenhouse study

	Mesocosm treatment	
Soil horizon (cm depth)	Well watered	Drought stressed
0–30	27.28	16.47
30–60	26.52	23.26
60–90	27.36	19.80
90–120	29.84	26.04
120–150	33.86	27.86

### Mesocosm sample collection and harvest measures

After flowering onset, the plants were destructively harvested 115 d after sowing and 87 d since the drought stress was applied. Phenotypic traits measured are defined in Table 2. One day before destructive sampling, plant height was recorded using a ruler, measured from the mesocosm medium surface to the tip of the tallest leaf when held to its maximum height, and all tillers were counted. Gas exchange and chlorophyll fluorescence parameters for the youngest fully expanded leaf of each plant were measured using an LI-6800 portable photosynthesis system with Multiphase Flash Fluorometer (LI-COR Biosciences, Lincoln, NE, USA) operating with a 6cm^2^ aperture circular leaf adaptor, a flow rate of 600 µmol mol^–1^, and a cuvette relative humidity of 60%. CO_2_ exchange was logged manually, with stability criteria for both ΔH_2_O and ΔCO_2_ SD limits set to 0.1 over a period of 15s. The leaf maximum width was used to normalize measurements on leaf material smaller than the circular leaf adaptor. Then, the mesocosms were injected with ^15^NO_3_ (98% atom) to assess deep N capture by switchgrass roots. Three evenly spaced passage holes were drilled around the circumference of each mesocosm at 132cm depth, and 5ml of Ca(^15^NO_3_)_2_ solution (0.46mg ^15^NO_3_ ml^−1^) was injected into each mesocosm through these holes with a syringe (Fig. 1B).

**Table 2.** Traits measured, descriptors, and methods used in this study

Trait category	Trait description	Method	Units
Total root size	Root dry mass total	Measured after 3 d at 60 °C	g per plant
	Root CO_2_ flux total	LI-8100A	nmol per plant s^–1^
	Root length total	RhizoVision Explorer	mm per plant
	Root length axial	RhizoVision Explorer	mm per plant
	Root length lateral	RhizoVision Explorer	mm per plant
	Root length secondary lateral	RhizoVision Explorer	mm per plant
	Root surface area total	RhizoVision Explorer	mm^2^ per plant
	Root surface area axial	RhizoVision Explorer	mm^2^ per plant
	Root surface area lateral	RhizoVision Explorer	mm^2^ per plant
	Root surface area secondary lateral	RhizoVision Explorer	mm^2^ per plant
	Root volume total	RhizoVision Explorer	mm^3^ per plant
	Root volume axial	RhizoVision Explorer	mm^3^ per plant
	Root volume lateral	RhizoVision Explorer	mm^3^ per plant
	Root volume secondary lateral	RhizoVision Explorer	mm^3^ per plant
	Root branch count total	RhizoVision Explorer	–
	Root tip count total	RhizoVision Explorer	–
Root distribution	Specific root length	Root length/root dry mass (derived)	m g^–1^
	Root lateral:axial ratio	Lateral+secondary lateral root length/crown root length (derived)	–
	Root branching frequency	RhizoVision Explorer	frequency mm^–1^
	Deep root dry mass total (mass basis)	Root dry mass in 120–150cm soil horizon	g per plant
	Deep root length total (length basis)	Root length in 120–150cm soil horizon	mm per plant
	Deep root fraction (mass basis)	Root dry mass in 120–150cm soil horizon/total root dry mass (derived)	g g^1^
	Deep root fraction (length basis)	Root length in 120–150cm soil horizon/total root length (derived)	mm mm^1^
Root diameter	Root maximum diameter	RhizoVision Explorer	mm per plant
	Root median diameter	RhizoVision Explorer	mm per plant
	Root average diameter	RhizoVision Explorer	mm per plant
Root respiration	Root CO_2_ flux total	LI-8100A	nmol per plant s^–1^
	Specific root CO_2_ flux (length basis)	LI-8100A and root length (derived)	nmol m^–1^ s^–1^
	Specific root CO_2_ flux (mass basis)	LI-8100A and root dry mass (derived)	nmol g^–1^ s^–1^
Biomass distribution	Plant dry mass total	Root+shoot dry mass (derived)	g per plant
	Root mass fraction	Root dry mass/total dry mass (derived)	g g^1^
Shoot size	Shoot dry mass total	Measured after 3 d at 60 °C	g per plant
	Plant height	Manual measurement soil level to leaf tip	cm per plant
	Leaf maximum width	Manual measurement widest leaf width	cm per plant
	Tiller count	Manual count	–
	Total leaf N	EA IRMS System	g per plant
Shoot properties	Leaf N concentration	EA IRMS System	%
	Leaf protein percent	EA IRMS System	%
	Leaf C concentration	EA IRMS System	%
	Net CO_2_ assimilation rate (A)	LI-6800	μmol m^–2^ s^1^
	Shoot ^15^N concentration	EA IRMS System	%
	Shoot ^15^N content	EA IRMS System	mg per plant
	Shoot ^15^N uptake rate	EA IRMS System	mg h^–1^ per plant
	Leaf transpiration rate (E)	LI-6800	mol m^–2^ s^1^
	Leaf stomatal conductance (gsw)	LI-6800	mol m^–2^ s^1^
	Intercellular CO_2_ partial pressure (Pci)	LI-6800	–

Calculations for derived traits are found in the associated R code. Traits measured on a per plant basis refer to the entire plant within one biological unit.

The next day at 24h after ^15^N injection, the shoot of each plant was severed at the stem base and dried at 60 °C for 3 d for dry biomass determination. The shoot samples for each plant were thoroughly ground by placing the samples into glass vials with three opposing surgical blades and shaking at a frequency of 30 Hz for 10min using a Qiagen TissueLyser II (Germantown, MD, USA). Shoot tissue percentages of total N and ^15^N were determined using a BioVisION from Elementar including an IsoPrime Vision isotope ratio mass spectrometer connected to an IsoPrime Isotope cube that operates in CNS mode (Elementar, Langenselbold, Germany).

For root harvesting, the polyethylene bag that lined each mesocosm was pulled out and the bag was sliced open longitudinally on a root washing station (Fig. 1C). A 100g aliquot of media mixture samples excluding roots was bagged at 30cm layers for measuring gravimetric water content and N content, as detailed above, placed in a cool box containing ice, and frozen at –20 °C within 8h. The rest of the mixture was then carefully washed away from the roots using a water hose with a low-pressure nozzle starting at the plant base. Immediately after root washing, roots were cut and divided into 30cm layers, and root respiration for each plant and layer was measured (Fig. 1D, E). All roots from each layer were transferred into a custom 43ml airtight chamber (as detailed in Guo *et al.*, 2021) connected to an LI-8100 Automated Soil CO_2_ Flux System (LI-COR Biosciences). A representative subsample of roots was measured if there was too much root material to fit into the chamber. The CO_2_ flux in the chamber was measured with an observation duration of 90s using the LI-8100A v4.0.9 software (Fig. 1D). The total respiration rate was calculated automatically by the linear fit mode in SoilFluxPro v4.0.1 software (LI-COR Biosciences) with a curve-fit time of 20–90s. After the root respiration measurement, the root material was bagged individually by plant, media layer, and by subsample if required (Fig. 1E). The root material was then placed in a cool box containing ice and frozen at –20 °C within 8h.

The bagged root samples were later thawed and imaged using a flatbed scanner equipped with a transparency unit (Epson Expression 12000XL, Epson America Inc, Los Alamitos, CA, USA). Roots were spread out on a transparent acrylic tray (420 mm×300mm) with a 5mm layer (400ml) of water and imaged at a resolution of 600 dpi as JPG files with 95% (high) quality. Multiple root scans were done when too much root material was present to scan in a single image to minimize root overlapping based on subjective determination, with an average root length of 10 m per scan retroactively calculated, and the cumulative root length was computed in R. Root tip count was slightly overestimated due to breaking of roots during sampling to depths, washing, and to fit into multiple scans. However, the number of lateral root tips far exceeds the number of broken root ends, so root tip count remains relevant. The axial, first-order lateral, and second-order lateral root lengths, surface area, and volume for each plant were calculated from the flatbed images using RhizoVision Explorer v2.0.2 (Seethepalli and York, 2020; Seethepalli *et al.*, 2021) based on diameter thresholds (in mm) of >0.9, 0.3–0.9, and <0.3, respectively. The diameter ranges were chosen based on tests with representative root scans from both genotypes, with the output images inspected to discover diameter ranges in which lateral roots typically were a different color from axial roots. The threshold level was set to 200, filter non-root objects set to 10mm^2^, and root pruning threshold set to 20 pixels. Total root tip number, branching frequencies, and average root diameter were also calculated in the software. During statistical analysis, the ratio of lateral roots (first- and second-order) to the axial traits was computed as lateral to axial ratios. After scanning, the root material was placed in a paper envelope and dried at 60 °C for 3 d for determination of root dry weight. Root mass fraction was calculated by dividing the total root dry mass by the total plant dry mass, and deep root fraction as the root length or mass in the bottom 120–150cm layer divided by the respective total root system length or mass.

### Statistical analysis

Statistical analyses were conducted using R version 4.0.3 (R Core Team, 2020); the statistical analysis R codes including the packages needed are available at the Zenodo Repository (https://doi.org/10.5281/zenodo.4281435; Griffiths *et al.*, 2021). Traits calculated are described in Table 2. ANOVA of the plant data was conducted using the R package ‘lmerTest’ (Kuznetsova *et al.*, 2017) with block as the random effect. The Tukey’s HSD test used for the multiple comparison boxplots was conducted using the R package ‘agricolae’ (De Mendiburu and Simon, 2015, Preprint). The correlation matrix was generated using the R package ‘corrplot’ (Wei and Simko, 2017) employing a pairwise Pearson correlation method for the plant trait data for both genotypes across all conditions. Linear discriminant analysis (LDA) was conducted using the ‘lda’ function from the MASS package (Venables and Ripley, 2002) to predict genotype, water treatment, or N treatment classes in separate analyses. Before LDA, data were standardized for each trait so that the mean was zero and the within-group SD was 1 in order to interpret loadings.

## Results

### Positive correlations of root size traits and deep rooting traits with shoot size traits in switchgrass

**Fig. 2.**
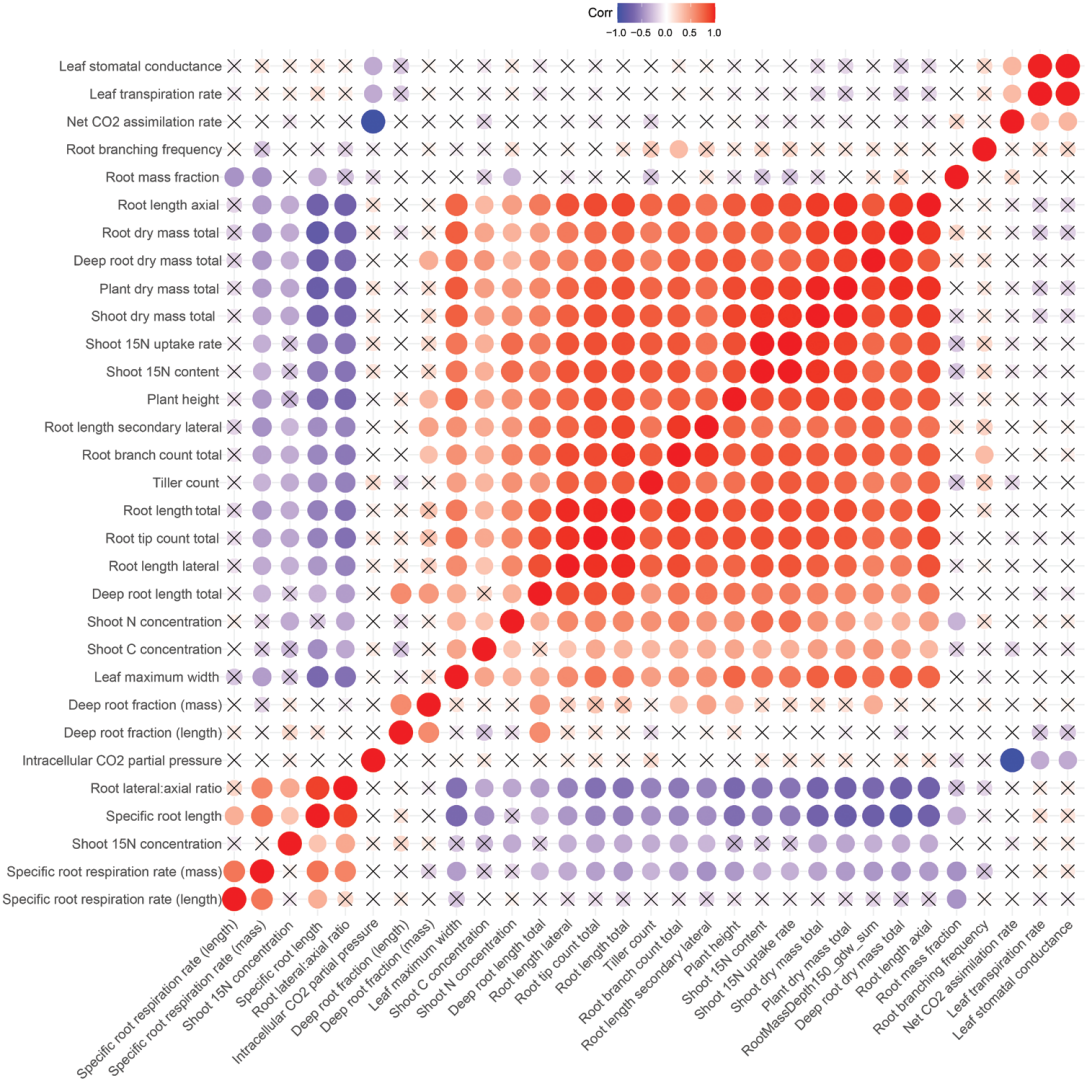
Correlation matrix for plant traits across both switchgrass ecotypes, upland (VS16) and lowland (AP13), and all conditions. Correlations are visualized using a color gradient. Red and blue colors represent a strong positive and negative correlation, respectively. No correlation is visualized with a cross symbol (*P*>0.05).

Across both switchgrass ecotypes and all conditions, a correlation matrix showed strong positive correlations among root size-related traits, deep rooting traits, shoot size-related traits, and ^15^N content of leaves (*P*<0.05, Fig. 2). Root length, surface area, and volume traits were highly correlated (*P*<0.05, Supplementary Fig. S1). For the specific root traits, positive correlations were observed between specific root length, specific root respiration (length and mass basis), and ^15^N percentage of leaves (*P*<0.05, Fig. 2). For the deep root fraction, the only correlated traits, aside from deep root mass, were for secondary lateral root traits and plant height (*P*<0.05, Fig. 2). Gas exchange (assimilation rate, transpiration rate, and stomatal conductance) and chlorophyll fluorescence parameters for the new fully expanded leaf were uncorrelated with all other measured plant traits (Fig. 2).

### Substantial phenotypic variation between the upland and lowland ecotypes for root and shoot traits representing the wide genetic pool of switchgrass

**Fig. 3.**
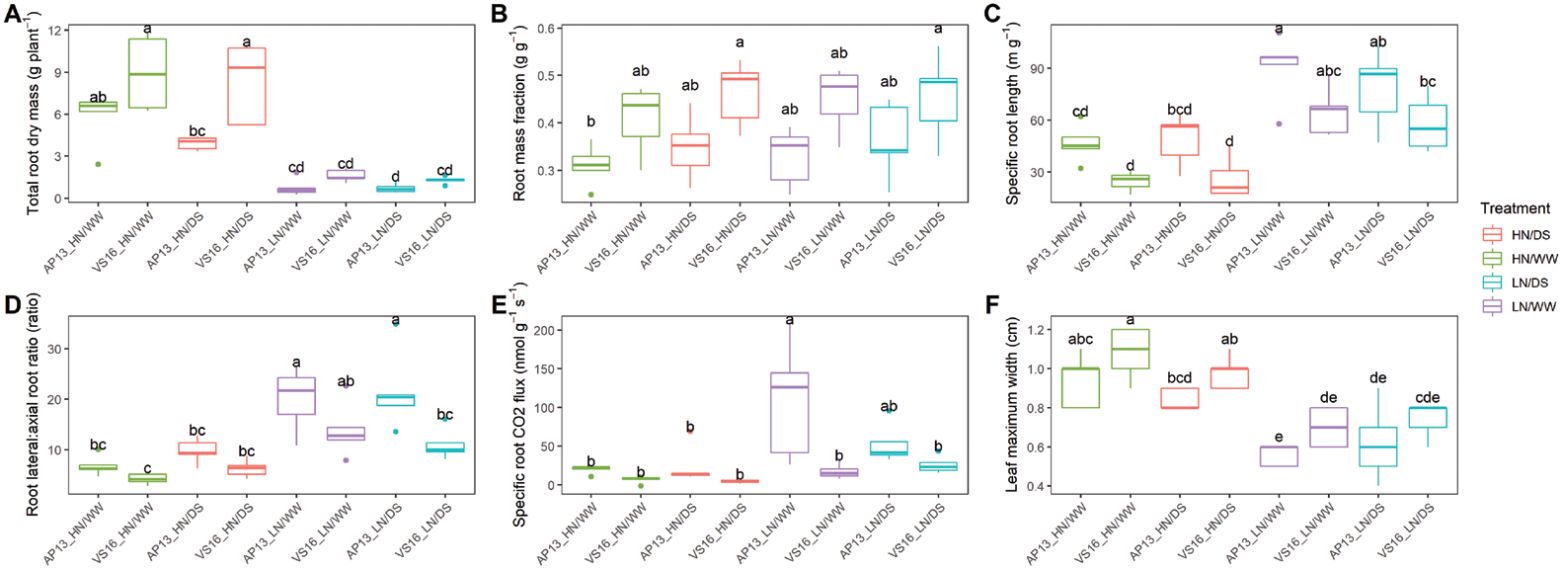
Total plant traits measured in each abiotic stress environment tested between the two switchgrass ecotypes, upland (VS16) and lowland (AP13). Boxes with the same letter were not significantly different at *P*<0.05 according to Tukey’s HSD test.

For the plant traits measured, substantial differences between the upland and lowland ecotypes were observed. Common to all conditions tested, total genotype-associated differences between the ecotypes were observed for total root mass, root class distribution traits including root mass fraction, specific root length, lateral:axial root ratio, and also specific root respiration rate (mass basis), plus leaf maximum width (*P*<0.05, Fig. 3; Supplementary Table S2). Across all conditions tested, the lowland ecotype had an average 80% larger root mass, 32% higher root mass fraction, 34% decrease in specific root length, 39% decrease in lateral:axial root ratio, and a 74% decrease in specific root respiration rate (mass basis) compared with the upland ecotype (Fig. 3; Supplementary Table S2). In favorable conditions only, namely HN/WW, genotypic differences were also observed for tiller count, with a 57% increase in the lowland ecotype (*P*<0.05, Supplementary Tables S3, S5). In all stress conditions tested (HN/DS, LN/WW, and LN/DS), genotypic differences were observed for root mass in the deepest mesocosm layer, with a 50% larger root mass in the lowland relative to the upland ecotype under HN conditions and a 140% larger root mass in the upland compared with the lowland ecotype under drought (*P*<0.05, Supplementary Tables S4–S6). Under the most severe stress condition, namely LN/DS, genotypic differences were observed for axial root size traits, with a 152% larger axial root system in the upland ecotype (length, surface area, volume) (*P*<0.001, Supplementary Tables S4, S6). Additional significant genotypic differences were observed in the LN/WW conditions for plant height, root branching frequency, root tip count, root length proportion in the deepest mesocosm layer compared with all layers, and total ^15^N content captured from the deepest layer, with the upland ecotype being larger for all (*P*<0.05, Supplementary Table S6).

**Fig. 4.**
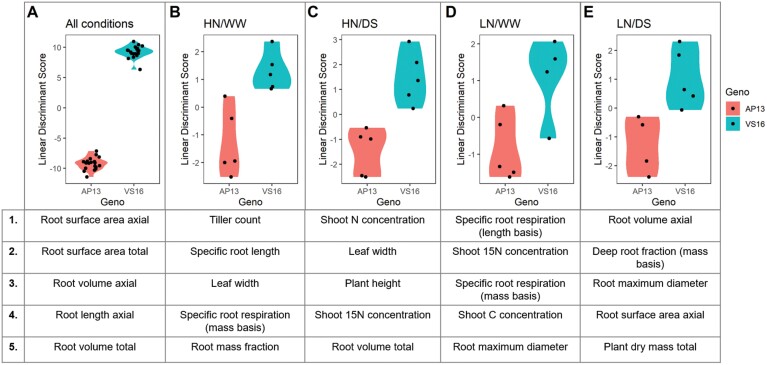
Linear discriminant analysis of total plant traits measured in each abiotic stress environment tested between the two switchgrass ecotypes, upland (VS16) and lowland (AP13). The five greatest discriminant traits by linear discriminant score, both positive and negative, are listed for each environment. (A) All conditions, (B) HN/WW, (C) HN/DS, (D) LN/WW, (E) LN/DS.

An LDA using the plant traits was performed to determine the differentiating capacity of the plant traits between the two ecotypes. Across all conditions, rooting traits were the greatest discriminators between the ecotypes, with axial and total root surface area being the greatest discriminants followed by root volume and length traits (Fig. 4A). In favorable conditions, namely HN/WW, the main discriminant traits were maximum tiller count, specific root length, leaf maximum width, specific root respiration rate (mass basis), and root mass fraction (Fig. 4B). Common to the well-watered conditions (HN/WW and LN/WW) was specific root respiration as a discriminant factor between the ecotypes (Fig. 4B, D). Common to the low N conditions (LN/WW and LN/DS) was root maximum diameter as a discriminant factor (Fig. 4D, E). Specific to HN/DS, the main discriminant factors between the switchgrass ecotypes were for shoot N and ^15^N concentration, and plant height (Fig. 4C). Shoot ^15^N concentration was also a main discriminant for LN/WW. Specific to LN/WW, specific root respiration rate (both mass and length basis) and shoot carbon concentration were the main discriminant traits (Fig. 4D). Specific to LN/DS, root mass at depth and dry mass total were the main discriminant traits (Fig. 4E).

### Switchgrass ecotypes share common plastic abiotic responses to N and water availability

Genotypic differences in phenotypic traits were observed between the upland and lowland ecotypes; however, in terms of abiotic stress responses, common plastic responses were also observed between the ecotypes.

A significant water treatment response was common to both switchgrass ecotypes (HN conditions), with larger axial root traits (length, surface area, and volume), lateral root traits (surface area andvolume), root tip count, root maximum diameter, and shoot traits (tiller count and maximum leaf width) in well-watered conditions relative to drought-stressed conditions (*P*<0.05, Supplementary Table S5). A significant increase in lateral:axial root ratio and deep root fraction (mass basis) was observed in the water stress conditions (HN conditions) (*P*<0.01, Supplementary Table S5). However, these water treatment effects were not observed in the LN conditions as the N stress appeared to have had a more severe and confounding effect on plant trait differences (Supplementary Table S6).

In response to N treatment, a significant treatment effect was observed for all plant size traits, with larger roots and shoots in HN conditions (WW and DS conditions, Supplementary Table S3, S4). In LN conditions, there was a significantly greater specific root length, lateral:axial root ratio, specific root respiration rate, and deep root fraction relative to the HN conditions. Traits with no significant difference by N treatment were for photosynthetic and transpiration measures (Supplementary Tables S3, S4). No significant difference in root mass fraction was observed for either N treatments or water treatments, with a shared reduction in both root and shoot mass by abiotic stress (Supplementary Tables S3, S4). Under well-watered conditions, a significant genotype by N treatment interaction was observed for secondary lateral root size traits, branching frequency, and specific root respiration rate (mass basis) (*P*<0.05, Supplementary Table S3). Under drought conditions, a significant genotype by N treatment interaction was observed for root dry mass total, axial root size traits, deep root mass total, and deep root fraction (*P*<0.05, Supplementary Table S4).

**Fig. 5.**
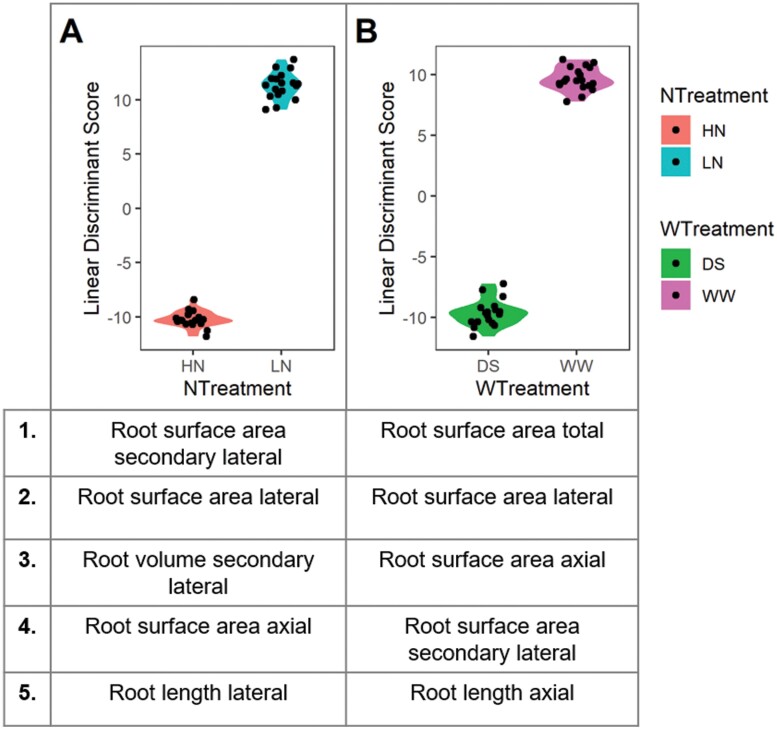
Linear discriminant analysis of total plant traits for both ecotypes to determine common discriminant traits by (A) N treatments and (B) water treatments. The five greatest discriminant traits by linear discriminant score, both positive and negative, are listed from all environment data.

An LDA using the plant traits was performed to determine the main discriminant trait between the water levels or N levels. The discriminant traits between N and water treatment levels were found to be rooting traits (Fig. 5A, B). For the N treatment, lateral and secondary lateral root traits were the top discriminant traits in addition to axial root surface area (Fig. 5A). For the water treatment, total root surface area and root surface area of each root class were the main discriminants, plus axial root length (Fig. 5B).

### Roots of both switchgrass ecotypes have the potential to grow deeper than 1.5 m with significant interaction between root depth-related traits and abiotic stress

Across both ecotypes, almost all rooting traits tested were significantly affected by depth (Supplementary Tables S7–S10). Exceptions were for specific root respiration, with no significant relationship with mesocosm depth and for lateral:axial root ratio in LN conditions. Roots for all plants reached the bottom of the 1.5 m mesocosm at 115 days after sowing.

Significant interactions between genotype and depth were observed for specific root respiration rate (weight basis) in favorable conditions, namely HN/WW. In the abiotic stress conditions, a significant genotype and depth interaction was observed for lateral root traits (length and surface area) in LN/WW, for root axial traits (length and surface area) and root average diameter in LN/DS, and for root maximum diameter in HN/DS.

**Fig. 6.**
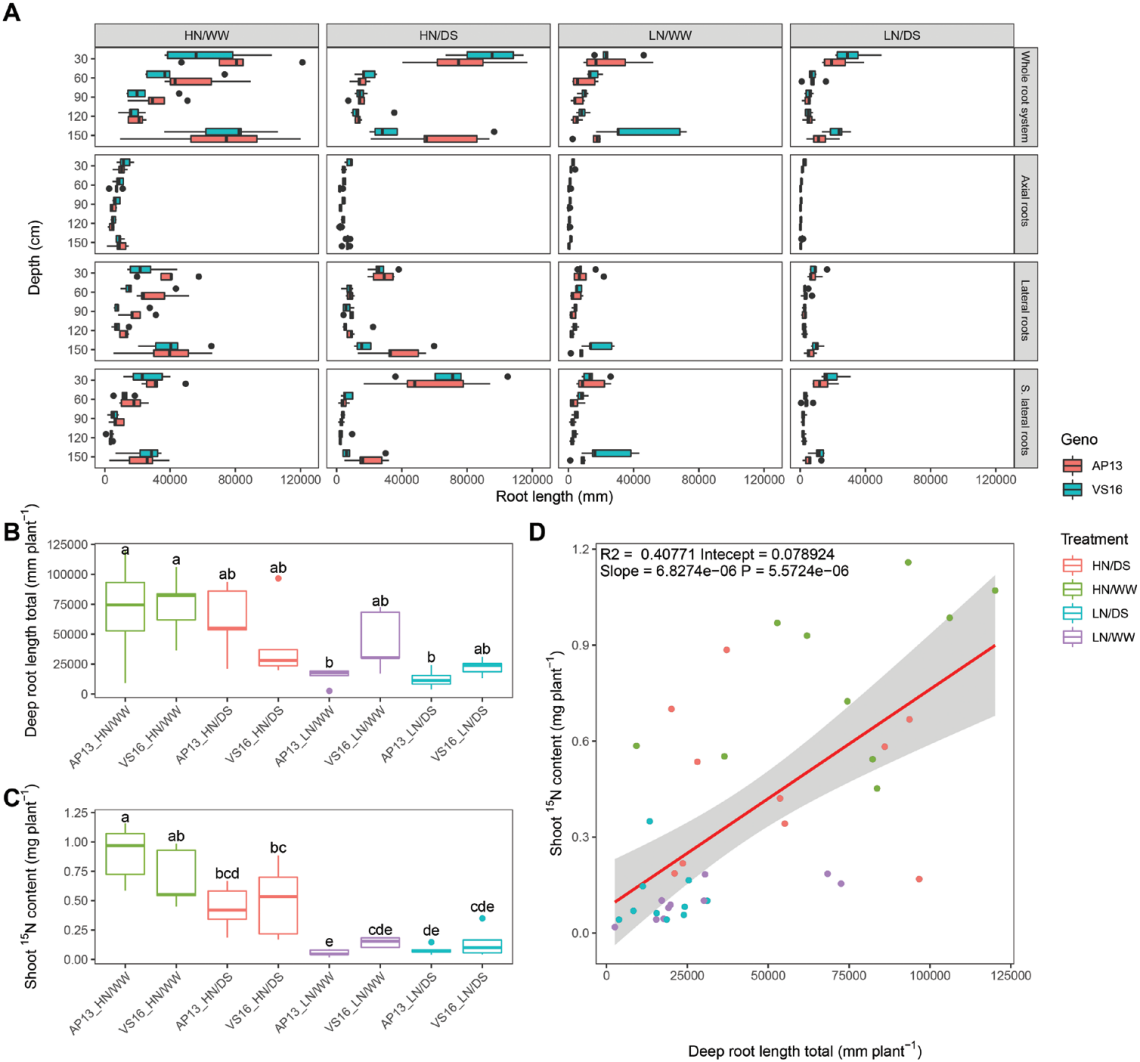
(A) Root distribution of upland (VS16) and lowland (AP13) switchgrass ecotypes across 1.5 m mesocosms under an abiotic stress environment. The root distributions by root class were separated into 30cm mesocosm layers. (B) Root length in the deepest layer and (C) ^15^N content in the shoot for the switchgrass ecotypes by treatment condition. Boxes with different letters were significantly different at *P*<0.05 according to Tukey’s HSD test. (D) Linear regression analysis using all data between root length in the deepest layer and ^15^N content in the shoot.

The root distribution across the vertical profile varied greatly by water and N conditions (Fig. 6A). For both switchgrass ecotypes, total root length was greatest in the favorable condition, HN/WW, and least in the combined stress condition, LN/DS. In favorable conditions, there was no significant genotypic difference in root length by layer. However, in the LN conditions, the upland ecotype had a greater root length in the deepest layer compared with the lowland ecotype (*P*<0.05, Supplementary Table S6). The greatest difference between the ecotypes was observed in the LN/WW condition, with the upland ecotype having a 193% increased root length in the deepest layer (*P*<0.05, Supplementary Table S6), with the LN/DS condition reducing the root length further at depth for both ecotypes. The differences observed in the root length by depth also agreed with the shoot ^15^N content results, with a 500% average greater ^15^N uptake in the HN condition than in the LN condition, reflecting uptake 24h after ^15^N was injected to the bottom layer (Fig. 6B, C, *P*<0.001; Supplementary Table S3). An increase in secondary lateral roots in the upland ecotype contributed to this root length increase in the deepest mesocosm layer (Fig. 6A, *P*<0.05; Supplementary Table S6). A positive significant relationship was observed between the root length in the deepest layer and ^15^N content in shoot material, with greater root length matching greater ^15^N uptake (*P*<0.001, Fig. 6D).

### Significant interaction between N and water stress combination treatments for axial and lateral root traits

Using the whole dataset containing both ecotypes and all conditions, interactions between N and water treatment were explored. Across all conditions, significant interactions between N and water treatment were observed for axial root traits (length, surface area, and volume), total root traits (volume and surface area), deep root fraction (mass basis) compared with all depths, shoot ^15^N content, ^15^N uptake rate, and shoot mass (*P*<0.05, Supplementary Table S11). In the lowland ecotype only, an interaction between N and water treatment was also observed for tiller count, total plant dry mass, and shoot N% (*P*<0.05, Supplementary Table S12). In the upland ecotype only, an interaction between N and water treatment was observed for secondary lateral root traits (length and surface area) (*P*<0.05, Supplementary Table S13).

## Discussion

Here, we show that members of two main ecotypes of switchgrass, upland (VS16) and lowland (AP13), share common root plastic response strategies to abiotic stress despite having large intrinsic root morphological differences. Appropriate growth responses to abiotic stress can be important stress mitigation strategies with efficient soil exploration for required resources. Despite previous studies finding switchgrass productivity of cultivar ‘Cave-in-Rock’ to be not receptive to fertilizer treatments (Duran *et al.*, 2016), here a large N treatment effect was found in both switchgrass ecotypes. Potentially, this cultivar could have a different N response, the field experiment could have been growth limited by other factors, or else the field soil had more residual available N than in the LN mesocosms. In response to N application in the current experiment, all plant size traits were significantly affected, with an overall reduction in root and shoot size traits under stress conditions (Supplementary Table S11). Similarly, water stress conditions in this study had significant plant size-reducing effects for both root and shoot traits (Supplementary Table S11).

The switchgrass root system makes up a large proportion of the plant biomass, with 34% of total biomass as roots for the lowland ecotype and 44% of total biomass in the upland ecotype in these single year plants, averaged across all treatments. Switchgrass can sequester a large amount of carbon and has been shown to increase soil carbon levels over time (Ma *et al.*, 2001). Interestingly, the differences observed in root mass fraction in this study were by ecotype only, with a stable fraction across water and N conditions (Supplementary Tables S2, S11). A significant interaction between drought and N stress conditions was observed in switchgrass for axial and lateral root traits, representing a complex and shared root development strategy for stress mitigation (Supplementary Table S11). Both ecotypes had a smaller axial and lateral root length in the stressed conditions compared with the favorable conditions, probably driven by a reduction in growth and photosynthate availability. A similar relationship was found across 12 temperate herbaceous species with changes in belowground biomass allocation in response to nutrient supply but no change in root mass fraction (Freschet *et al.*, 2015). For switchgrass, the main discriminants between favorable and stress conditions, water and N, across both ecotypes were for total root surface area size traits (Fig. 5). Roots are a large carbon investment and maintenance cost to the plant, and therefore a reduced root axial investment under stressed conditions is an efficient plastic root response. These innate responses to abiotic stress reflect the hardiness of the species and the ideal nature of switchgrass as a low-input crop (Vogel, 1996).

Specifically, in response to N conditions, lateral root traits were also found to be the main discriminants (Fig. 5). Lateral roots are regarded as the primary site for the uptake of soil resources and often the greatest contributor to total root length and surface area in contact with soil (Hund *et al.*, 2009; Yu *et al.*, 2019). Total root length was reduced under N stress, indicating that the plant was unable to sustain the total carbon cost of a large root system. However, the inability to maintain the larger total size was partially compensated by more efficient carbon use by increasing the allocation to cheaper lateral roots, as shown by the greater lateral:axial root ratio (Fig. 3). An increase in resource distribution to lateral roots, therefore, increased the root exploration in the soil with a reduced resource allocation to roots, which can be seen as an efficient abiotic stress adaptive response, as also shown in maize (Guo and York, 2019). This highlights the importance of lateral roots for abiotic stress mitigation in switchgrass and that selection for improved, resource-efficient switchgrass varieties could use the lateral:axial root ratio as a selection criterion for further investigation. This trait is convenient as it can be measured in a subsample of the root system, rather than requiring full excavation or measurements of entire root systems. Increased lateral rooting relative to axial rooting would also be an efficient way to increase the contact of root deposition with soil minerals to encourage carbon sequestration in deep soil layers (Poirier *et al.*, 2018).

Switchgrass can be found across a wide range of climatic conditions, and the upland and lowland ecotype represent the main divergent groups. Members of each ecotype, AP13 and VS16, were chosen for this study as AP13 is the source of the lowland reference genome and mapping populations have been derived from crossing the two ecotypes (Milano *et al.*, 2016). Variation among these ecotypes and others could be harnessed for improving abiotic stress tolerance and yield. Between these two switchgrass ecotypes, large morphological differences were observed in root traits, with potential implications for abiotic stress tolerance (Fig. 4). In a previous study, the upland ecotype was found to be more drought tolerant and had higher N demand than the lowland ecotype in 1 gallon pot trials, but the root traits were not quantified (Milano *et al.*, 2016). In this single year study, a greater root mass was found in the upland ecotype in all conditions, which may be a contributor to its greater drought tolerance potential, although a shoot biomass difference was not observed. Across all conditions, rooting traits were the greatest discriminant between the ecotypes, with lateral and secondary lateral roots being the main discriminants by N condition (Figs 4, 5). The two ecotypes did not differ by shoot mass per condition in this study, but there was a significant tiller count difference, with the lowland ecotype having a greater number of tillers in favorable conditions (HN/WW) (Supplementary Table S3, S5). Tiller counts in switchgrass have been shown to vary greatly year by year in field trials which is a likely response to the environment including rainfall patterns and competition with neighboring plants (Cassida *et al.*, 2005; Price and Casler, 2014). The upland ecotype maintained the same number of tillers between the N and water conditions, indicating stability across abiotic stress. The lowland ecotype tillered more in favorable conditions which may translate to an increase in overall shoot biomass and resilience across multiple years (Supplementary Table S3, S5). Therefore, the upland and lowland ecotypes have varying strategies and adaptations that may translate to stress resistance and productivity in varying environments. Upland alleles have been previously associated with shoot size and vigor, which may explain the greater root mass differences observed between the ecotypes in this study (Lowry *et al.*, 2019).

An important plant trait for water and nutrient capture is deep rooting; however, it is technically challenging to excavate a representative root system from the field and quantify root length by soil depths. In this study, 1.5 m mesocosms were used to phenotype root distribution in switchgrass in 30cm layers along the vertical profile with minimal root loss compared with field studies. Switchgrass is a particularly deep-rooted species and, in this study, a positive correlation was found between root length at depth and deep ^15^N capture by roots (Fig. 6). Both ecotypes had roots in the deepest layer and had the potential to grow deeper than 1.5 m, given the substantial root length density in the bottom layers after only 115 d of growth (Fig. 6). Deep rooting is an important trait for crop performance because water and nitrate are often found in deep soil layers. Variance observed in switchgrass root size traits and ^15^N capture were found to explain differences in shoot mass, highlighting the link between root and shoot (Fig. 6). Between the upland and lowland ecotypes, differences in abiotic stress mitigation strategies were observed. Across all stress conditions tested, a genotype-associated difference was observed between the upland and lowland ecotypes for root mass in the deepest layer of the mesocosm (Supplementary Tables S4, S6). In the LN conditions, a greater root length and mass were observed in the upland ecotype which conformed to a greater ^15^N shoot content which was applied to the deepest layer (Supplementary Table S6). Therefore, the upland ecotype was more receptive to the vertical N stress gradient with greater root development at depth and greater N uptake, which is an advantageous trait for low input cropping systems. Given the difficulties in excavating root systems and soil coring in the field, injection of ^15^N in deep layers and measuring uptake in the shoot is a viable method to screen for deep rooting activity. Designing taller mesocosms would allow for more precise evaluation of deep rooting and deep nutrient capture for these switchgrass ecotypes. Interestingly, in response to drought conditions, the lowland ecotype had a smaller root mass compared with the upland ecotype in the deepest layer; however, the upland ecotype had a greater proportion of roots in this bottom layer (Fig. 6). This indicates a root length distribution change to the vertical gradient water stress in the lowland ecotype and could be an advantageous drought tolerance trait.

Our findings highlight the importance of the root system, with switchgrass ecotypes sharing common strategies for abiotic stress mitigation and deep N capture. We also show that the ecotypes have differing strategies to abiotic stress tolerance with biomass distribution changes and deep rooting in response to factorial water and N stress. Admixture between the divergent genomes is expected to enhance climate adaptation and yield improvement (Lovell *et al.*, 2021). For switchgrass to be a productive bioenergy crop, a balance between productivity and resource sustainability will have to be reached by enhancing plant abiotic stress tolerance and soil resource use efficiency while potentially sequestering soil carbon.

## Supplementary data

The following supplementary data are available at *JXB* online.

Table S1. Monthly average greenhouse conditions for the study.

Table S2. ANOVA for plant phenotypic traits as influenced by switchgrass ecotype across all conditions.

Table S3. ANOVA for plant phenotypic traits as influenced by switchgrass ecotype and N condition under well-watered conditions (LN/WW and HN/WW data).

Table S4. ANOVA for plant phenotypic traits as influenced by switchgrass ecotype and N condition under drought conditions (LN/DS and HN/DS data).

Table S5. ANOVA for plant phenotypic traits as influenced by switchgrass ecotype and water condition under HN conditions (HN/WW and HN/DS data).

Table S6. ANOVA for plant phenotypic traits as influenced by switchgrass ecotype and water condition under LN conditions (LN/WW and LN/DS data).

Table S7. ANOVA for plant phenotypic traits as influenced by switchgrass ecotype and soil depth under HN/WW conditions.

Table S8. ANOVA for plant phenotypic traits as influenced by switchgrass ecotype and soil depth under LN/WW conditions.

Table S9. ANOVA for plant phenotypic traits as influenced by switchgrass ecotype and soil depth under HN/DS conditions.

Table S10. ANOVA for plant phenotypic traits as influenced by switchgrass ecotype and soil depth under LN/DS conditions.

Table S11. ANOVA for plant phenotypic traits as influenced by N and water conditions (both ecotypes).

Table S12. ANOVA for plant phenotypic traits as influenced by N and water conditions in the lowland ecotype (AP13).

Table S13. ANOVA for plant phenotypic traits as influenced by N and water conditions in the upland ecotype (VS16).

erab437_suppl_Supplementary_Tables_S1-S13

## Data Availability

All the data and R statistical code required for reproducing the statistics and figures used in this paper are publicly available at https://doi.org/10.5281/zenodo.4281435 (Griffiths *et al.*, 2021).
