# Supplementary material for: Interactions among rooting traits for deep water and nitrogen uptake in upland and lowland ecotypes of switchgrass (Panicum virgatum L.)
Source: J Exp Bot. 2021 Oct 4;73(3):967–79. doi: 10.1093/jxb/erab437 (PMC8793874; doi:10.1093/jxb/erab437)
Supplement: erab437_suppl_Supplementary_Tables_S1-S13 [file erab437_suppl_supplementary_tables_s1-s13.pdf]

Table S1. Monthly average greenhouse conditions for study.

Month	Average monthly temperature (°C)		Average monthly PAR (μmol m^{-2} s^{-1})
	Day (15h)	Night (9h)	
October	24.87	19.92	151.06
November	24.22	18.60	141.40
December	23.83	18.22	150.02
January	23.56	18.27	144.60

Table S2. Analysis of variance (ANOVA) for plant phenotypic traits as influenced by switchgrass ecotype across all conditions.

	Traits	Source of variation
		Geno
Total root size	Root dry mass total (g $plant^{-1}$)	4.25 *
	Root length total (mm $plant^{-1}$)	0.05 ns
	Root length axial (mm $plant^{-1}$)	1.50 ns
	Root length lateral (mm $plant^{-1}$)	0.25 ns
	Root length secondary lateral (mm $plant^{-1}$)	0.65 ns
	Root surface area total (mm^2 $plant^{-1}$)	0.21 ns
	Root surface area axial (mm^2 $plant^{-1}$)	2.09 ns
	Root surface area lateral (mm^2 $plant^{-1}$)	0.42 ns
	Root surface area secondary lateral (mm^2 $plant^{-1}$)	0.66 ns
	Root volume total (mm^3 $plant^{-1}$)	1.06 ns
	Root volume axial (mm^3 $plant^{-1}$)	2.54 ns
	Root volume lateral (mm^3 $plant^{-1}$)	0.59 ns
	Root volume secondary lateral (mm^3 $plant^{-1}$)	0.66 ns
	Root branch count total	0.00 ns
	Root tip count total	0.03 ns
Root distribution	Specific root length (m g^{-1})	8.83 **
	Root lateral:axial root ratio (ratio)	6.96 *
	Root branching frequency (branch mm^{-1})	0.73 ns
	Deep root mass total (g $plant^{-1}$)	2.82 ns
	Deep root length total (mm $plant^{-1}$)	0.29 ns
	Deep root mass fraction (g g^{1})	0.80 ns
	Deep root length fraction (mm mm^{1})	2.60 ns
Root diameter	Root diameter mean (mm $plant^{-1}$)	4.29 *
	Root diameter maximum (mm $plant^{-1}$)	0.10 ns
	Root diameter median (mm $plant^{-1}$)	3.89 ns
Root respiration	Root CO_2 flux total (nmol $plant^{-1}$ s^{-1})	8.24 **
	Specific root CO_2 flux (nmol g^{-1} s^{-1})	8.68 **
	Specific root CO_2 flux (nmol m^{-1} s^{-1})	4.47 *
Biomass distribution	Root mass fraction (g g^{1})	31.50 ***
	Total plant mass (g $plant^{-1}$)	1.28 ns
Shoot size	Shoot dry mass total (g $plant^{-1}$)	0.25 ns
	Plant height (cm $plant^{-1}$)	0.81 ns
	Tiller count	1.80 ns
	Leaf maximum width (cm)	4.69 *
Shoot properties	Leaf carbon content (%)	1.78 ns
	Leaf N concentration (%)	2.07 ns
	Leaf 15N concentration (%)	1.09 ns
	Leaf total 15N content (mg $plant^{-1}$)	0.00 ns
	Leaf 15N uptake rate (mg $plant^{-1}$ h^{-1})	0.00 ns
	CO_2 assimilation rate (µmol m^{-2} s^{-1})	0.98 ns
	Transpiration rate (mol m^{-2} s^{-1})	0.02 ns
	Stomatal conductance (mol m^{-2} s^{-1})	0.01 ns
	Intracellular CO_2 (Pci)	0.58 ns

*** $P < 0.001$; ** $P < 0.01$; * $P < 0.05$; ns not significant

Table S3. Analysis of variance (ANOVA) for plant phenotypic traits as influenced by switchgrass ecotype and N condition under well-watered conditions (LN/WW and HN/WW data).

	Traits	Source of variation		
		Geno	N Treatment	Geno:N Treatment
Total root size	Root dry mass total (g $plant^{-1}$)	7.31 *	69.17 ***	2.40 ns
	Root length total (mm $plant^{-1}$)	0.01 ns	35.47 ***	2.55 ns
	Root length axial (mm $plant^{-1}$)	1.66 ns	62.92 ***	0.16 ns
	Root length lateral (mm $plant^{-1}$)	0.39 ns	31.93 ***	2.63 ns
	Root length secondary lateral (mm $plant^{-1}$)	0.59 ns	17.62 ***	4.89 *
	Root surface area total (mm^2 $plant^{-1}$)	0.15 ns	53.14 ***	0.69 ns
	Root surface area axial (mm^2 $plant^{-1}$)	3.06 ns	76.61 ***	0.78 ns
	Root surface area lateral (mm^2 $plant^{-1}$)	0.58 ns	31.93 ***	2.78 ns
	Root surface area secondary lateral (mm^2 $plant^{-1}$)	0.59 ns	18.85 ***	4.55 *
	Root volume total (mm^3 $plant^{-1}$)	1.50 ns	78.42 ***	0.08 ns
	Root volume axial (mm^3 $plant^{-1}$)	4.32 ns	85.96 ***	1.68 ns
	Root volume lateral (mm^3 $plant^{-1}$)	0.76 ns	31.54 ***	2.90 ns
	Root volume secondary lateral (mm^3 $plant^{-1}$)	0.57 ns	19.91 ***	4.27 ns
	Root branch count total	0.45 ns	25.89 ***	4.02 ns
	Root tip count total	0.01 ns	72.03 ***	4.19 ns
Root distribution	Specific root length (m g^{-1})	20.87 ***	65.28 ***	0.14 ns
	Root lateral:axial root ratio (ratio)	5.85 *	40.26 ***	0.96 ns
	Root branching frequency (branch mm^{-1})	0.05 ns	0.06 ns	6.05 *
	Deep root mass total (g $plant^{-1}$)	3.39 ns	44.87 ***	0.02 ns
	Deep root length total (mm $plant^{-1}$)	1.73 ns	11.67 **	0.97 ns
	Deep root mass fraction (g g^{1})	1.16 ns	0.15 ns	1.80 ns
	Deep root length fraction (mm mm^{1})	4.87 *	0.21 ns	0.13 ns
Root diameter	Root diameter mean (mm $plant^{-1}$)	9.84 **	113.07 ***	2.58 ns
	Root diameter maximum (mm $plant^{-1}$)	4.56 ns	52.39 ***	5.38 *
	Root diameter median (mm $plant^{-1}$)	0.13 ns	125.29 ***	0.22 ns
Root respiration	Root CO_2 flux total (nmol $plant^{-1}$ s^{-1})	19.10 **	21.07 ***	3.33 ns
	Specific root CO_2 flux (nmol g^{-1} s^{-1})	8.43 *	7.59 *	4.73 *
	Specific root CO_2 flux (nmol m^{-1} s^{-1})	4.05 ns	1.10 ns	2.04 ns
Biomass distribution	Root mass fraction (g g^{1})	16.01 **	1.18 ns	0.20 ns
	Total plant mass (g $plant^{-1}$)	2.22 ns	105.19 ***	0.45 ns
Shoot size	Shoot dry mass total (g $plant^{-1}$)	0.20 ns	101.06 ***	0.00 ns
	Plant height (cm $plant^{-1}$)	1.31 ns	155.83 ***	7.59 *
	Tiller count	6.63 *	34.61 ***	3.38 ns
	Leaf maximum width (cm)	12.38 **	91.20 ***	0.00 ns
Shoot properties	Leaf carbon content (%)	1.11 ns	46.12 ***	1.79 ns
	Leaf N concentration (%)	2.85 ns	24.57 ***	5.40 *
	Leaf 15N concentration (%)	6.02 *	7.28 *	5.95 *
	Leaf total 15N content (mg $plant^{-1}$)	0.59 ns	81.88 ***	3.79 ns
	Leaf 15N uptake rate (mg $plant^{-1}$ h^{-1})	0.59 ns	81.88 ***	3.79 ns
	CO_2 assimilation rate (µmol m^{-2} s^{-1})	0.39 ns	0.30 ns	0.49 ns
	Transpiration rate (mol m^{-2} s^{-1})	0.84 ns	0.64 ns	2.85 ns
	Stomatal conductance (mol m^{-2} s^{-1})	1.17 ns	0.46 ns	2.88 ns
	Intracellular CO_2 (Pci)	0.30 ns	0.31 ns	0.15 ns

*** P < 0.001; ** P < 0.01; * P < 0.05; ns not significant

Table S4. Analysis of variance (ANOVA) for plant phenotypic traits as influenced by switchgrass ecotype and N condition under drought conditions (LN/DS and HN/DS data).

	Traits	Source of variation		
		Geno	N Treatment	Geno:N Treatment
Total root size	Root dry mass total (g $plant^{-1}$)	15.24 **	65.39 ***	8.85 *
	Root length total (mm $plant^{-1}$)	0.42 ns	60.89 ***	0.24 ns
	Root length axial (mm $plant^{-1}$)	24.57 ***	180.80 ***	5.43 *
	Root length lateral (mm $plant^{-1}$)	0.42 ns	40.14 ***	1.52 ns
	Root length secondary lateral (mm $plant^{-1}$)	1.01 ns	32.96 ***	0.00 ns
	Root surface area total (mm^2 $plant^{-1}$)	2.53 ns	125.47 ***	0.00 ns
	Root surface area axial (mm^2 $plant^{-1}$)	31.27 ***	177.18 ***	11.09 **
	Root surface area lateral (mm^2 $plant^{-1}$)	0.91 ns	44.15 ***	2.24 ns
	Root surface area secondary lateral (mm^2 $plant^{-1}$)	1.09 ns	33.86 ***	0.02 ns
	Root volume total (mm^3 $plant^{-1}$)	20.73 ***	236.90 ***	6.46 *
	Root volume axial (mm^3 $plant^{-1}$)	29.51 ***	136.14 ***	14.01 **
	Root volume lateral (mm^3 $plant^{-1}$)	1.56 ns	48.61 ***	3.21 ns
	Root volume secondary lateral (mm^3 $plant^{-1}$)	1.15 ns	34.27 ***	0.04 ns
	Root branch count total	0.74 ns	42.15 ***	0.01 ns
	Root tip count total	0.19 ns	52.23 ***	2.15 ns
Root distribution	Specific root length (m g^{-1})	8.32 *	16.88 ***	0.03 ns
	Root lateral:axial root ratio (ratio)	12.46 **	17.24 ***	3.12 ns
	Root branching frequency (branch mm^{-1})	1.83 ns	0.00 ns	0.03 ns
	Deep root mass total (g $plant^{-1}$)	11.10 **	87.74 ***	5.39 *
	Deep root length total (mm $plant^{-1}$)	0.31 ns	11.99 **	2.44 ns
	Deep root mass fraction (g g^1)	0.09 ns	8.96 **	2.46 ns
	Deep root length fraction (mm mm^1)	0.08 ns	0.09 ns	5.39 *
Root diameter	Root diameter mean (mm $plant^{-1}$)	17.25 **	82.15 ***	0.41 ns
	Root diameter maximum (mm $plant^{-1}$)	20.18 ***	54.57 ***	0.90 ns
	Root diameter median (mm $plant^{-1}$)	0.25 ns	26.80 ***	0.00 ns
Root respiration	Root CO_2 flux total (nmol $plant^{-1}$ s^{-1})	4.16 ns	2.63 ns	2.98 ns
	Specific root CO_2 flux (nmol g^{-1} s^{-1})	6.86 *	8.09 *	0.17 ns
	Specific root CO_2 flux (nmol m^{-1} s^{-1})	0.66 ns	0.05 ns	1.20 ns
Biomass distribution	Root mass fraction (g g^1)	13.77 **	0.01 ns	0.15 ns
	Total plant mass (g $plant^{-1}$)	6.41 *	71.82 ***	3.57 ns
Shoot size	Shoot dry mass total (g $plant^{-1}$)	1.64 ns	57.43 ***	0.84 ns
	Plant height (cm $plant^{-1}$)	2.10 ns	27.21 ***	0.01 ns
	Tiller count	0.04 ns	57.85 ***	0.04 ns
	Leaf maximum width (cm)	8.59 *	26.90 ***	0.05 ns
Shoot properties	Leaf carbon content (%)	4.92 *	30.35 ***	2.10 ns
	Leaf N concentration (%)	1.05 ns	3.67 ns	0.07 ns
	Leaf 15N concentration (%)	0.07 ns	4.92 *	0.07 ns
	Leaf total 15N content (mg $plant^{-1}$)	0.52 ns	17.37 ***	0.00 ns
	Leaf 15N uptake rate (mg $plant^{-1}$ h^{-1})	0.52 ns	17.37 ***	0.00 ns
	CO_2 assimilation rate (µmol m^{-2} s^{-1})	0.54 ns	0.00 ns	1.46 ns
	Transpiration rate (mol m^{-2} s^{-1})	1.16 ns	0.26 ns	0.00 ns
	Stomatal conductance (mol m^{-2} s^{-1})	1.45 ns	0.34 ns	0.00 ns
	Intracellular CO_2 (Pci)	0.25 ns	0.00 ns	0.75 ns

*** P < 0.001; ** P < 0.01; * P < 0.05; ns not significant

Table S5. Analysis of variance (ANOVA) for plant phenotypic traits as influenced by switchgrass ecotype and water condition under HN conditions (HN/WW and HN/DS data).

Traits		Source of variation		
		Geno	W Treatment	Geno:W Treatment
Total root size	Root dry mass total (g plant^{-1})	14.99 **	1.72 ns	0.37 ns
	Root length total (mm plant^{-1})	0.41 ns	2.26 ns	0.54 ns
	Root length axial (mm plant^{-1})	3.83 ns	13.63 **	0.11 ns
	Root length lateral (mm plant^{-1})	2.24 ns	5.28 *	0.28 ns
	Root length secondary lateral (mm plant^{-1})	0.01 ns	1.20 ns	0.90 ns
	Root surface area total (mm^2 plant^{-1})	0.01 ns	9.14 **	0.29 ns
	Root surface area axial (mm^2 plant^{-1})	7.58 *	16.57 ***	0.06 ns
	Root surface area lateral (mm^2 plant^{-1})	2.88 ns	5.61 *	0.26 ns
	Root surface area secondary lateral (mm^2 plant^{-1})	0.03 ns	0.16 ns	0.86 ns
	Root volume total (mm^3 plant^{-1})	3.30 ns	17.29 ***	0.13 ns
	Root volume axial (mm^3 plant^{-1})	11.18 **	18.53 ***	0.04 ns
	Root volume lateral (mm^3 plant^{-1})	3.49 ns	5.90 *	0.25 ns
	Root volume secondary lateral (mm^3 plant^{-1})	0.04 ns	0.01 ns	0.81 ns
	Root branch count total	0.60 ns	0.01 ns	1.62 ns
	Root tip count total	2.30 ns	5.45 *	0.04 ns
Root distribution	Specific root length (m g^{-1})	22.31 ***	0.18 ns	0.00 ns
	Root lateral:axial root ratio (ratio)	17.04 **	11.25 **	0.40 ns
	Root branching frequency (branch mm^{-1})	0.14 ns	0.29 ns	3.19 ns
	Deep root mass total (g plant^{-1})	7.28 *	1.03 ns	1.37 ns
	Deep root length total (mm plant^{-1})	0.32 ns	1.96 ns	0.72 ns
	Deep root mass fraction (g g^{1})	0.66 ns	8.87 **	0.27 ns
	Deep root length fraction (mm mm^{1})	0.00 ns	0.76 ns	4.23 ns
Root diameter	Root diameter mean (mm plant^{-1})	12.84 **	3.41 ns	0.03 ns
	Root diameter maximum (mm plant^{-1})	3.36 ns	7.02 *	4.47 ns
	Root diameter median (mm plant^{-1})	0.21 ns	0.24 ns	0.00 ns
Root respiration	Root CO_2 flux total (nmol plant^{-1} s^{-1})	8.39 *	0.18 ns	0.74 ns
	Specific root CO_2 flux (nmol g^{-1} s^{-1})	7.74 *	0.02 ns	0.29 ns
	Specific root CO_2 flux (nmol m^{-1} s^{-1})	2.34 ns	0.10 ns	0.18 ns
Biomass distribution	Root mass fraction (g g^{1})	21.69 ***	4.08 ns	0.13 ns
	Total plant mass (g plant^{-1})	5.30 *	6.13 *	0.41 ns
Shoot size	Shoot dry mass total (g plant^{-1})	0.82 ns	8.45 *	0.32 ns
	Plant height (cm plant^{-1})	0.05 ns	3.90 ns	0.99 ns
	Tiller count	4.90 *	6.60 *	4.15 ns
	Leaf maximum width (cm)	12.12 **	6.19 *	0.00 ns
Shoot properties	Shoot carbon content (%)	0.34 ns	60.52 ***	1.06 ns
	Shoot N concentration (%)	4.42 ns	2.16 ns	0.62 ns
	Shoot 15N concentration (%)	0.01 ns	3.63 ns	0.07 ns
	Shoot total 15N content (mg plant^{-1})	0.44 ns	8.54 **	1.47 ns
	Shoot 15N uptake rate (mg plant^{-1} h^{-1})	0.44 ns	8.54 **	1.47 ns
	CO_2 assimilation rate (µmol m^{-2} s^{-1})	0.14 ns	0.22 ns	0.09 ns
	Transpiration rate (mol m^{-2} s^{-1})	0.40 ns	1.09 ns	2.30 ns
	Stomatal conductance (mol m^{-2} s^{-1})	0.44 ns	0.79 ns	2.75 ns
	Intracellular CO_2 (Pci)	0.02 ns	0.40 ns	0.09 ns

*** P < 0.001; ** P < 0.01; * P < 0.05; ns not significant

Table S6. Analysis of variance (ANOVA) for plant phenotypic traits as influenced by switchgrass ecotype and water condition under LN conditions (LN/WW and LN/DS data).

	Traits	Source of variation		
		Geno	W Treatment	Geno:W Treatment
Total root size	Root dry mass total (g $plant^{-1}$)	14.15 **	0.97 ns	0.50 ns
	Root length total (mm $plant^{-1}$)	7.52 *	2.60 ns	1.17 ns
	Root length axial (mm $plant^{-1}$)	17.73 ***	1.55 ns	0.01 ns
	Root length lateral (mm $plant^{-1}$)	4.42 ns	2.54 ns	1.10 ns
	Root length secondary lateral (mm $plant^{-1}$)	7.98 *	2.40 ns	1.34 ns
	Root surface area total (mm^2 $plant^{-1}$)	10.22 **	2.82 ns	1.00 ns
	Root surface area axial (mm^2 $plant^{-1}$)	18.72 ***	1.11 ns	0.03 ns
	Root surface area lateral (mm^2 $plant^{-1}$)	4.33 ns	2.66 ns	1.17 ns
	Root surface area secondary lateral (mm^2 $plant^{-1}$)	8.23 *	2.66 ns	1.37 ns
	Root volume total (mm^3 $plant^{-1}$)	16.18 ***	2.24 ns	0.63 ns
	Root volume axial (mm^3 $plant^{-1}$)	17.15 ***	0.64 ns	0.06 ns
	Root volume lateral (mm^3 $plant^{-1}$)	4.36 ns	2.80 ns	1.15 ns
	Root volume secondary lateral (mm^3 $plant^{-1}$)	8.38 *	2.83 ns	1.37 ns
	Root branch count total	7.16 *	1.23 ns	0.51 ns
	Root tip count total	8.43 *	1.85 ns	1.16 ns
Root distribution	Specific root length (m g^{-1})	8.02 *	1.44 ns	0.12 ns
	Root lateral:axial root ratio (ratio)	10.09 **	0.06 ns	0.73 ns
	Root branching frequency (branch mm^{-1})	1.74 ns	0.08 ns	0.00 ns
	Deep root mass total (g $plant^{-1}$)	17.73 ***	1.23 ns	1.63 ns
	Deep root length total (mm $plant^{-1}$)	9.54 **	3.53 ns	2.28 ns
	Deep root mass fraction (g g^{1})	3.99 ns	0.27 ns	0.11 ns
	Deep root length fraction (mm mm^{1})	5.84 *	1.54 ns	0.43 ns
Root diameter	Root diameter mean (mm $plant^{-1}$)	9.00 **	0.09 ns	1.06 ns
	Root diameter maximum (mm $plant^{-1}$)	14.01 **	0.00 ns	0.04 ns
	Root diameter median (mm $plant^{-1}$)	0.30 ns	1.32 ns	0.54 ns
Root respiration	Root CO_2 flux total (nmol $plant^{-1}$ s^{-1})	4.50 ns	0.95 ns	0.64 ns
	Specific root CO_2 flux (nmol g^{-1} s^{-1})	8.47 *	1.37 ns	2.54 ns
	Specific root CO_2 flux (nmol m^{-1} s^{-1})	2.43 ns	0.51 ns	2.92 ns
Biomass distribution	Root mass fraction (g g^{1})	10.22 **	0.33 ns	0.20 ns
	Total plant mass (g $plant^{-1}$)	8.25 *	1.56 ns	0.30 ns
Shoot size	Shoot dry mass total (g $plant^{-1}$)	3.20 ns	1.67 ns	0.12 ns
	Plant height (cm $plant^{-1}$)	18.66 ***	4.35 ns	0.30 ns
	Tiller count	0.63 ns	0.16 ns	0.63 ns
	Leaf maximum width (cm)	8.11 *	1.20 ns	0.05 ns
Shoot properties	Shoot carbon content (%)	6.87 *	10.61 **	0.77 ns
	Shoot N concentration (%)	0.04 ns	0.04 ns	0.67 ns
	Shoot 15N concentration (%)	1.34 ns	1.07 ns	0.43 ns
	Shoot total 15N content (mg $plant^{-1}$)	5.95 *	0.14 ns	0.20 ns
	Shoot 15N uptake rate (mg $plant^{-1}$ h^{-1})	5.95 *	0.14 ns	0.20 ns
	CO_2 assimilation rate (µmol m^{-2} s^{-1})	1.97 ns	0.01 ns	0.17 ns
	Transpiration rate (mol m^{-2} s^{-1})	1.14 ns	1.19 ns	0.06 ns
	Stomatal conductance (mol m^{-2} s^{-1})	1.13 ns	1.19 ns	0.14 ns
	Intracellular CO_2 (Pci)	1.06 ns	0.00 ns	0.05 ns

*** P < 0.001; ** P < 0.01; * P < 0.05; ns not significant

Table S7. Analysis of variance (ANOVA) for plant phenotypic traits as influenced by switchgrass ecotype and soil depth under HN/WW conditions.

	Traits	Source of variation		
		Geno	Depth	Geno:Depth
Total root size	Root dry mass (g $plant^{-1}$)	14.99 ***	17.59 ***	0.56 ns
	Root length total (mm $plant^{-1}$)	13.47 ***	1.74 ns	0.45 ns
	Root length axial (mm $plant^{-1}$)	7.18 ***	2.87 ns	0.28 ns
	Root length lateral (mm $plant^{-1}$)	11.05 ***	4.69 *	0.77 ns
	Root length secondary lateral (mm $plant^{-1}$)	16.90 ***	1.01 ns	0.49 ns
	Root surface area total (mm^2 $plant^{-1}$)	11.26 ***	0.18 ns	0.12 ns
	Root surface area axial (mm^2 $plant^{-1}$)	7.99 ***	6.99 *	0.36 ns
	Root surface area lateral (mm^2 $plant^{-1}$)	10.72 ***	5.60 *	0.73 ns
	Root surface area secondary lateral (mm^2 $plant^{-1}$)	15.66 ***	0.93 ns	0.44 ns
	Root volume total (mm^3 $plant^{-1}$)	10.09 ***	2.15 ns	0.02 ns
	Root volume axial (mm^3 $plant^{-1}$)	8.81 ***	11.84 **	0.43 ns
	Root volume lateral (mm^3 $plant^{-1}$)	10.40 ***	6.36 *	0.67 ns
	Root volume secondary lateral (mm^3 $plant^{-1}$)	14.93 ***	0.87 ns	0.41 ns
	Root branch count total	19.50 ***	5.93 *	0.70 ns
	Root tip count total	15.87 ***	2.02 ns	0.85 ns
Root distribution	Specific root length (m g^{-1})	11.43 ***	24.81 ***	0.88 ns
	Root lateral:axial root ratio (ratio)	5.07 **	14.04 ***	2.20 ns
	Root branching frequency (branch mm^{-1})	70.38 ***	19.01 ***	0.63 ns
Root diameter	Root diameter mean (mm $plant^{-1}$)	16.82 ***	34.32 ***	2.61 ns
	Root diameter maximum (mm $plant^{-1}$)	2.78 *	2.23 ns	0.91 ns
	Root diameter median (mm $plant^{-1}$)	13.59 ***	1.74 ns	0.90 ns
Root respiration	Specific root CO_2 flux (nmol g^{-1} s^{-1})	1.94 ns	44.89 ***	2.92 *
	Specific root CO_2 flux (nmol m^{-1} s^{-1})	0.76 ns	2.95 ns	1.27 ns

*** P < 0.001; ** P < 0.01; * P < 0.05; ns not significant

Table S8. Analysis of variance (ANOVA) for plant phenotypic traits as influenced by switchgrass ecotype and soil depth under LN/WW conditions.

	Traits	Source of variation		
		Geno	Depth	Geno:Depth
Total root size	Root dry mass (g $plant^{-1}$)	12.88 ***	16.13 ***	0.46 ns
	Root length total (mm $plant^{-1}$)	8.75 ***	7.62 **	2.55 ns
	Root length axial (mm $plant^{-1}$)	20.23 ***	21.97 ***	0.50 ns
	Root length lateral (mm $plant^{-1}$)	8.28 ***	6.26 *	2.65 *
	Root length secondary lateral (mm $plant^{-1}$)	8.18 ***	6.85 *	2.56 ns
	Root surface area total (mm^2 $plant^{-1}$)	9.60 ***	10.26 **	2.12 ns
	Root surface area axial (mm^2 $plant^{-1}$)	22.86 ***	26.55 ***	0.73 ns
	Root surface area lateral (mm^2 $plant^{-1}$)	8.53 ***	6.28 *	2.66 *
	Root surface area secondary lateral (mm^2 $plant^{-1}$)	7.70 ***	7.37 **	2.50 ns
	Root volume total (mm^3 $plant^{-1}$)	12.74 ***	16.73 ***	1.31 ns
	Root volume axial (mm^3 $plant^{-1}$)	23.61 ***	29.14 ***	1.08 ns
	Root volume lateral (mm^3 $plant^{-1}$)	8.53 ***	6.39 *	2.54 ns
	Root volume secondary lateral (mm^3 $plant^{-1}$)	7.37 ***	7.76 **	2.46 ns
	Root branch count total	11.30 ***	5.15 *	2.49 ns
	Root tip count total	10.24 ***	8.15 **	2.28 ns
Root distribution	Specific root length (m g^{-1})	4.87 **	9.74 **	0.96 ns
	Root lateral:axial root ratio (ratio)	1.18 ns	4.70 *	1.28 ns
	Root branching frequency (branch mm^{-1})	15.79 ***	2.29 ns	0.10 ns
Root diameter	Root diameter mean (mm $plant^{-1}$)	1.84 ns	7.38 *	0.99 ns
	Root diameter maximum (mm $plant^{-1}$)	5.82 **	15.60 ***	0.11 ns
	Root diameter median (mm $plant^{-1}$)	2.08 ns	0.00 ns	0.63 ns
Root respiration	Specific root CO_2 flux (nmol g^{-1} s^{-1})	1.41 ns	8.10 **	0.79 ns
	Specific root CO_2 flux (nmol m^{-1} s^{-1})	1.40 ns	7.68 *	0.38 ns

*** P < 0.001; ** P < 0.01; * P < 0.05; ns not significant

Table S9. Analysis of variance (ANOVA) for plant phenotypic traits as influenced by switchgrass ecotype and soil depth under HN/DS conditions.

	Traits	Source of variation		
		Geno	Depth	Geno:Depth
Total root size	Root dry mass (g $plant^{-1}$)	10.65 ***	66.52 ***	0.70 ns
	Root length total (mm $plant^{-1}$)	28.56 ***	0.01 ns	0.99 ns
	Root length axial (mm $plant^{-1}$)	22.42 ***	38.21 ***	2.11 ns
	Root length lateral (mm $plant^{-1}$)	16.88 ***	1.75 ns	0.87 ns
	Root length secondary lateral (mm $plant^{-1}$)	35.93 ***	0.24 ns	0.88 ns
	Root surface area total (mm^2 $plant^{-1}$)	25.61 ***	0.91 ns	1.25 ns
	Root surface area axial (mm^2 $plant^{-1}$)	20.41 ***	63.06 ***	1.56 ns
	Root surface area lateral (mm^2 $plant^{-1}$)	17.33 ***	2.95 ns	1.01 ns
	Root surface area secondary lateral (mm^2 $plant^{-1}$)	34.02 ***	0.22 ns	0.90 ns
	Root volume total (mm^3 $plant^{-1}$)	26.77 ***	20.61 ***	1.09 ns
	Root volume axial (mm^3 $plant^{-1}$)	16.65 ***	75.58 ***	0.79 ns
	Root volume lateral (mm^3 $plant^{-1}$)	17.93 ***	4.43 *	1.19 ns
	Root volume secondary lateral (mm^3 $plant^{-1}$)	32.08 ***	0.21 ns	0.90 ns
	Root branch count total	36.95 ***	0.16 ns	0.72 ns
	Root tip count total	30.44 ***	1.58 ns	1.97 ns
Root distribution	Specific root length (m g^{-1})	20.79 ***	27.77 ***	1.68 ns
	Root lateral:axial root ratio (ratio)	30.01 ***	17.67 ***	1.18 ns
	Root branching frequency (branch mm^{-1})	110.23 ***	0.01 ns	0.63 ns
Root diameter	Root diameter mean (mm $plant^{-1}$)	25.17 ***	21.99 ***	1.63 ns
	Root diameter maximum (mm $plant^{-1}$)	6.22 ***	10.30 **	4.27 **
	Root diameter median (mm $plant^{-1}$)	10.13 ***	0.91 ns	0.24 ns
Root respiration	Specific root CO_2 flux (nmol g^{-1} s^{-1})	0.56 ns	7.09 *	0.55 ns
	Specific root CO_2 flux (nmol m^{-1} s^{-1})	0.22 ns	2.67 ns	0.64 ns

*** P < 0.001; ** P < 0.01; * P < 0.05; ns not significant

Table S10. Analysis of variance (ANOVA) for plant phenotypic traits as influenced by switchgrass ecotype and soil depth under LN/DS conditions.

	Traits	Source of variation		
		Geno	Depth	Geno:Depth
Total root size	Root dry mass (g $plant^{-1}$)	42.18 ***	26.92 ***	1.08 ns
	Root length total (mm $plant^{-1}$)	23.14 ***	4.44 *	1.59 ns
	Root length axial (mm $plant^{-1}$)	33.12 ***	45.52 ***	2.96 *
	Root length lateral (mm $plant^{-1}$)	15.41 ***	2.26 ns	1.32 ns
	Root length secondary lateral (mm $plant^{-1}$)	25.76 ***	3.54 ns	1.71 ns
	Root surface area total (mm^2 $plant^{-1}$)	26.39 ***	9.61 **	1.83 ns
	Root surface area axial (mm^2 $plant^{-1}$)	33.98 ***	47.91 ***	2.78 *
	Root surface area lateral (mm^2 $plant^{-1}$)	15.44 ***	2.01 ns	1.28 ns
	Root surface area secondary lateral (mm^2 $plant^{-1}$)	22.51 ***	3.74 ns	1.63 ns
	Root volume total (mm^3 $plant^{-1}$)	35.23 ***	26.14 ***	2.27 ns
	Root volume axial (mm^3 $plant^{-1}$)	28.72 ***	41.28 ***	2.04 ns
	Root volume lateral (mm^3 $plant^{-1}$)	14.85 ***	2.03 ns	1.16 ns
	Root volume secondary lateral (mm^3 $plant^{-1}$)	20.34 ***	3.91 ns	1.57 ns
	Root branch count total	38.75 ***	4.93 *	2.25 ns
	Root tip count total	23.74 ***	4.90 *	1.47 ns
Root distribution	Specific root length (m g^{-1})	15.51 ***	44.68 ***	2.50 ns
	Root lateral:axial root ratio (ratio)	0.89 ns	2.67 ns	0.91 ns
	Root branching frequency (branch mm^{-1})	33.31 ***	0.77 ns	2.06 ns
Root diameter	Root diameter mean (mm $plant^{-1}$)	4.39 **	31.80 ***	2.69 *
	Root diameter maximum (mm $plant^{-1}$)	6.39 ***	14.91 ***	1.90 ns
	Root diameter median (mm $plant^{-1}$)	8.52 ***	2.31 ns	1.09 ns
Root respiration	Specific root CO_2 flux (nmol g^{-1} s^{-1})	5.56 **	20.81 ***	0.82 ns
	Specific root CO_2 flux (nmol m^{-1} s^{-1})	9.61 ***	0.01 ns	0.38 ns

*** P < 0.001; ** P < 0.01; * P < 0.05; ns not significant

Table S11. Analysis of variance (ANOVA) for plant phenotypic traits as influenced by N condition and W condition (both ecotypes).

	Traits	Source of variation		
		N Treat	W Treat	N Treat:W Treat
Total root size	Root dry mass total (g $plant^{-1}$)	75.02 ***	1.25 ns	0.69 ns
	Root length total (mm $plant^{-1}$)	85.10 ***	3.97 ns	0.64 ns
	Root length axial (mm $plant^{-1}$)	119.47 ***	13.13 ***	10.01 **
	Root length lateral (mm $plant^{-1}$)	62.18 ***	6.73 *	3.00 ns
	Root length secondary lateral (mm $plant^{-1}$)	48.07 ***	0.04 ns	2.77 ns
	Root surface area total (mm^2 $plant^{-1}$)	127.79 ***	11.91 **	6.40 *
	Root surface area axial (mm^2 $plant^{-1}$)	119.28 ***	13.19 ***	10.90 **
	Root surface area lateral (mm^2 $plant^{-1}$)	60.91 ***	6.82 *	3.30 ns
	Root surface area secondary lateral (mm^2 $plant^{-1}$)	48.98 ***	0.20 ns	1.28 ns
	Root volume total (mm^3 $plant^{-1}$)	154.84 ***	17.23 ***	13.08 ***
	Root volume axial (mm^3 $plant^{-1}$)	110.74 ***	12.59 **	11.13 **
	Root volume lateral (mm^3 $plant^{-1}$)	59.16 ***	6.88 *	3.51 ns
	Root volume secondary lateral (mm^3 $plant^{-1}$)	49.60 ***	0.84 ns	0.54 ns
	Root branch count total	56.71 ***	0.17 ns	0.05 ns
	Root tip count total	112.60 ***	6.64 *	2.61 ns
Root distribution	Specific root length (m g^{-1})	38.11 ***	0.43 ns	1.00 ns
	Root lateral:axial root ratio (ratio)	33.92 ***	0.30 ns	0.84 ns
	Root branching frequency (branch mm^{-1})	0.02 ns	0.28 ns	0.01 ns
	Deep root mass total (g $plant^{-1}$)	90.11 ***	0.38 ns	1.10 ns
	Deep root length total (mm $plant^{-1}$)	22.27 ***	3.95 ns	0.29 ns
	Deep root mass fraction (g g^{1})	3.00 ns	2.31 ns	5.29 *
	Deep root length fraction (mm mm^{1})	0.26 ns	1.87 ns	0.03 ns
Root diameter	Root diameter mean (mm $plant^{-1}$)	102.59 ***	1.28 ns	1.91 ns
	Root diameter maximum (mm $plant^{-1}$)	58.09 ***	1.09 ns	1.13 ns
	Root diameter median (mm $plant^{-1}$)	108.52 ***	0.01 ns	0.74 ns
Root respiration	Root CO_2 flux total (nmol $plant^{-1}$ s^{-1})	5.57 *	0.22 ns	0.04 ns
	Specific root CO_2 flux (nmol g^{-1} s^{-1})	9.89 **	1.40 ns	1.56 ns
	Specific root CO_2 flux (nmol m^{-1} s^{-1})	1.25 ns	0.39 ns	0.81 ns
Biomass distribution	Root mass fraction (g g^{1})	0.39 ns	1.52 ns	0.25 ns
	Total plant mass (g $plant^{-1}$)	146.09 ***	5.84 *	4.03 ns
Shoot size	Shoot dry mass total (g $plant^{-1}$)	175.36 ***	10.40 **	7.69 **
	Plant height (cm $plant^{-1}$)	94.07 ***	6.25 *	1.13 ns
	Tiller count	49.23 ***	4.64 *	3.52 ns
	Leaf maximum width (cm)	62.54 ***	0.42 ns	3.78 ns
Shoot properties	Shoot carbon content (%)	59.84 ***	26.63 ***	0.05 ns
	Shoot N concentration (%)	18.04 ***	1.03 ns	1.56 ns
	Shoot 15N concentration (%)	9.49 **	1.02 ns	1.14 ns
	Shoot total 15N content (mg $plant^{-1}$)	82.85 ***	7.36 *	8.48 **
	Shoot 15N uptake rate (mg $plant^{-1}$ h^{-1})	82.85 ***	7.36 *	8.48 **
	CO_2 assimilation rate (μmol m^{-2} s^{-1})	0.10 ns	0.03 ns	0.13 ns
	Transpiration rate (mol m^{-2} s^{-1})	0.78 ns	2.24 ns	0.03 ns
	Stomatal conductance (mol m^{-2} s^{-1})	0.70 ns	1.81 ns	0.00 ns
	Intracellular CO_2 (Pci)	0.17 ns	0.19 ns	0.13 ns

*** $P < 0.001$; ** $P < 0.01$; * $P < 0.05$; ns not significant

Table S12. Analysis of variance (ANOVA) for plant phenotypic traits as influenced by N condition and W condition in the lowland ecotype (AP13).

	Traits	Source of variation		
		N Treat	W Treat	N Treat:W Treat
Total root size	Root dry mass total (g $plant^{-1}$)	90.55 ***	4.85 *	4.33 ns
	Root length total (mm $plant^{-1}$)	50.73 ***	2.24 ns	1.53 ns
	Root length axial (mm $plant^{-1}$)	54.55 ***	8.50 *	6.76 *
	Root length lateral (mm $plant^{-1}$)	60.64 ***	4.92 *	3.94 ns
	Root length secondary lateral (mm $plant^{-1}$)	26.72 ***	0.00 ns	0.04 ns
	Root surface area total (mm^2 $plant^{-1}$)	63.57 ***	6.00 *	4.85 *
	Root surface area axial (mm^2 $plant^{-1}$)	62.14 ***	10.54 **	9.09 *
	Root surface area lateral (mm^2 $plant^{-1}$)	60.49 ***	4.94 *	4.05 ns
	Root surface area secondary lateral (mm^2 $plant^{-1}$)	29.11 ***	0.18 ns	0.02 ns
	Root volume total (mm^3 $plant^{-1}$)	71.60 ***	10.29 **	9.14 *
	Root volume axial (mm^3 $plant^{-1}$)	68.40 ***	13.03 **	12.01 **
	Root volume lateral (mm^3 $plant^{-1}$)	58.86 ***	4.87 *	4.00 ns
	Root volume secondary lateral (mm^3 $plant^{-1}$)	30.93 ***	0.50 ns	0.15 ns
	Root branch count total	29.40 ***	0.71 ns	0.50 ns
	Root tip count total	68.76 ***	2.30 ns	1.93 ns
Root distribution	Specific root length (m g^{-1})	22.23 ***	0.43 ns	0.91 ns
	Root lateral:axial root ratio (ratio)	28.37 ***	0.95 ns	0.08 ns
	Root branching frequency (branch mm^{-1})	0.54 ns	0.11 ns	0.37 ns
	Deep root mass total (g $plant^{-1}$)	39.11 ***	0.01 ns	0.02 ns
	Deep root length total (mm $plant^{-1}$)	20.02 ***	0.20 ns	0.06 ns
	Deep root mass fraction (g g^{1})	6.26 *	1.95 ns	2.51 ns
	Deep root length fraction (mm mm^{1})	0.74 ns	0.14 ns	0.98 ns
Root diameter	Root diameter mean (mm $plant^{-1}$)	109.74 ***	2.84 ns	0.92 ns
	Root diameter maximum (mm $plant^{-1}$)	60.80 ***	2.11 ns	2.83 ns
	Root diameter median (mm $plant^{-1}$)	93.70 ***	0.15 ns	0.41 ns
Root respiration	Root CO_2 flux total (nmol $plant^{-1}$ s^{-1})	8.01 *	0.49 ns	0.41 ns
	Specific root CO_2 flux (nmol g^{-1} s^{-1})	8.85 **	1.74 ns	2.32 ns
	Specific root CO_2 flux (nmol m^{-1} s^{-1})	1.87 ns	0.91 ns	1.95 ns
Biomass distribution	Root mass fraction (g g^{1})	0.39 ns	2.04 ns	0.00 ns
	Total plant mass (g $plant^{-1}$)	94.45 ***	7.20 *	6.10 *
Shoot size	Shoot dry mass total (g $plant^{-1}$)	87.73 ***	7.74 *	6.44 *
	Plant height (cm $plant^{-1}$)	43.91 ***	3.74 ns	1.68 ns
	Tiller count	34.24 ***	7.74 *	4.87 *
	Leaf maximum width (cm)	35.76 ***	0.16 ns	2.54 ns
Shoot properties	Shoot carbon content (%)	23.36 ***	25.20 ***	0.00 ns
	Shoot N concentration (%)	38.71 ***	1.54 ns	6.34 *
	Shoot 15N concentration (%)	1.65 ns	1.26 ns	1.33 ns
	Shoot total 15N content (mg $plant^{-1}$)	75.30 ***	9.83 **	12.29 **
	Shoot 15N uptake rate (mg $plant^{-1}$ h^{-1})	75.30 ***	9.83 **	12.29 **
	CO_2 assimilation rate (μmol m^{-2} s^{-1})	0.77 ns	0.00 ns	0.51 ns
	Transpiration rate (mol m^{-2} s^{-1})	0.03 ns	0.01 ns	0.34 ns
	Stomatal conductance (mol m^{-2} s^{-1})	0.04 ns	0.01 ns	0.44 ns
	Intracellular CO_2 (Pci)	0.16 ns	0.11 ns	0.33 ns

*** $P < 0.001$; ** $P < 0.01$; * $P < 0.05$; ns not significant

Table S13. Analysis of variance (ANOVA) for plant phenotypic traits as influenced by N condition and water condition in the upland ecotype (VS16).

	Traits	Source of variation		
		N Treat	W Treat	N Treat:W Treat
Total root size	Root dry mass total (g plant^{-1})	67.50 ***	0.33 ns	0.04 ns
	Root length total (mm plant^{-1})	34.40 ***	1.73 ns	0.05 ns
	Root length axial (mm plant^{-1})	86.85 ***	6.76 *	4.91 *
	Root length lateral (mm plant^{-1})	15.61 **	2.53 ns	0.40 ns
	Root length secondary lateral (mm plant^{-1})	25.17 ***	0.19 ns	7.55 *
	Root surface area total (mm^2 plant^{-1})	57.75 ***	5.30 *	1.54 ns
	Root surface area axial (mm^2 plant^{-1})	100.54 ***	7.56 *	5.96 *
	Root surface area lateral (mm^2 plant^{-1})	15.44 **	2.72 ns	0.55 ns
	Root surface area secondary lateral (mm^2 plant^{-1})	23.38 ***	0.04 ns	4.81 *
	Root volume total (mm^3 plant^{-1})	101.18 ***	8.79 **	5.52 *
	Root volume axial (mm^3 plant^{-1})	103.67 ***	7.63 *	6.44 *
	Root volume lateral (mm^3 plant^{-1})	15.34 **	2.93 ns	0.70 ns
	Root volume secondary lateral (mm^3 plant^{-1})	21.96 ***	0.40 ns	3.14 ns
	Root branch count total	33.49 ***	0.31 ns	2.39 ns
	Root tip count total	54.98 ***	6.28 *	0.90 ns
Root distribution	Specific root length (m g^{-1})	42.07 ***	0.22 ns	0.60 ns
	Root lateral:axial root ratio (ratio)	24.67 ***	0.08 ns	2.83 ns
	Root branching frequency (branch mm^{-1})	0.97 ns	1.24 ns	0.62 ns
	Deep root mass total (g plant^{-1})	100.41 ***	1.51 ns	4.32 ns
	Deep root length total (mm plant^{-1})	5.08 *	6.21 *	0.29 ns
	Deep root mass fraction (g g^{1})	0.05 ns	0.53 ns	2.96 ns
	Deep root length fraction (mm mm^{1})	2.05 ns	5.01 *	0.32 ns
Root diameter	Root diameter mean (mm plant^{-1})	72.34 ***	0.39 ns	2.13 ns
	Root diameter maximum (mm plant^{-1})	28.99 ***	0.10 ns	0.01 ns
	Root diameter median (mm plant^{-1})	37.98 ***	0.01 ns	0.37 ns
Root respiration	Root CO_2 flux total (nmol plant^{-1} s^{-1})	1.22 ns	0.06 ns	1.92 ns
	Specific root CO_2 flux (nmol g^{-1} s^{-1})	20.54 ***	0.80 ns	2.42 ns
	Specific root CO_2 flux (nmol m^{-1} s^{-1})	0.05 ns	2.40 ns	2.87 ns
Biomass distribution	Root mass fraction (g g^{1})	0.38 ns	1.06 ns	0.77 ns
	Total plant mass (g plant^{-1})	87.53 ***	1.75 ns	0.85 ns
Shoot size	Shoot dry mass total (g plant^{-1})	75.48 ***	2.86 ns	1.77 ns
	Plant height (cm plant^{-1})	72.51 ***	3.30 ns	0.00 ns
	Tiller count	39.02 ***	0.10 ns	0.39 ns
	Leaf maximum width (cm)	62.00 ***	0.58 ns	3.16 ns
Shoot properties	Shoot carbon content (%)	40.72 ***	7.70 *	0.13 ns
	Shoot N concentration (%)	3.88 ns	0.40 ns	0.01 ns
	Shoot 15N concentration (%)	9.89 **	0.07 ns	0.09 ns
	Shoot total 15N content (mg plant^{-1})	24.24 ***	1.11 ns	1.05 ns
	Shoot 15N uptake rate (mg plant^{-1} h^{-1})	24.24 ***	1.11 ns	1.05 ns
	CO_2 assimilation rate (μmol m^{-2} s^{-1})	1.52 ns	0.06 ns	0.01 ns
	Transpiration rate (mol m^{-2} s^{-1})	2.46 ns	4.84 *	0.88 ns
	Stomatal conductance (mol m^{-2} s^{-1})	2.43 ns	5.05 *	0.78 ns
	Intracellular CO_2 (Pci)	0.93 ns	0.10 ns	0.00 ns

*** P < 0.001; ** P < 0.01; * P < 0.05; ns not significant
